# Histone H3 posttranslational modified enzymes defined neutrophil plasticity and their vulnerability to IL-10 in the course of the inflammation

**DOI:** 10.1186/s12950-024-00389-8

**Published:** 2024-05-14

**Authors:** Paweł Piatek, Magdalena Namiecinska, Natalia Lewkowicz, Małgorzata Kulińska-Michalska, Zbigniew Jabłonowski, Mariola Matysiak, Sylwia Michlewska, Marek Wieczorek, Przemysław Lewkowicz

**Affiliations:** 1 Department of Immunogenetics, Medical University of Lodz, ul. Pomorska 251/A4, 92- 213 Lodz, Poland; 2 Department of Periodontology and Oral Mucosal Diseases, Medical University of Lodz, 90-419 Lodz, Poland; 3 Department of Urology, Medical University of Lodz, 90-647 Lodz, Poland; 4 Department of Neurology, Medical University of Lodz, 90-153 Lodz, Poland; 5 Laboratory of Microscopic Imaging and Specialized Biological Techniques, Faculty of Biology and Environmental Protection, University of Lodz, 90-237 Lodz, Poland; 6 Department of Neurobiology, Faculty of Biology and Environmental Protection, University of Lodz, 90-236 Lodz, Poland

**Keywords:** Neutrophils, Posttranslational histone modification, H3K4me3-marked histone, Periodontitis, Sepsis, Neuromyelitis Optica Spectrum disorders

## Abstract

**Background:**

Neutrophils are a heterogeneous population capable of antimicrobial functions associated with pre-activation/activation and tissue regeneration. The specific polarisation of immune cells is mediated by the modification of ‘chromatin landscapes’, which enables differentiated access and activity of regulatory elements that guarantee their plasticity during inflammation No specific pattern within histone posttranslational modifications (PTMs) controlling this plasticity has been identified.

**Methods:**

Using the in vitro model of inflammation, reflecting different states of neutrophils from resting, pre-activated cells to activated and reducing tissue regeneration, we have analysed 11 different histone posttranslational modifications (PTMs), PTM enzymes associated with remodelling neutrophil chromatin, and H3K4me3 ChIP-Seq Gene Ontology analysis focusing on the processes related to histone PTMs. These findings were verified by extrapolation to adequate clinical status, using neutrophils derived from the patients with sepsis (systemic septic inflammation with LPS-stimulated neutrophils), neuromyelitis optical spectrum disorders (aseptic inflammation with pre-activated neutrophils) and periodontitis (local self-limiting septic inflammation with IL-10-positive neutrophils).

**Results:**

Physiological activation of neutrophils comprises a pre-activation characterised by histone H3K27ac and H3K4me1, which position enhancers; direct LPS exposure is induced explicitly by H3K4me3 which marked Transcription Start Site (TSS) regions and low-level of H3K9me3, H3K79me2 and H3K27me3 which, in turn, marked repressed genes. Contrary to antimicrobial action, IL-10 positively induced levels of H3S10p and negatively H3K9me3, which characterised processes related to the activation of genes within heterochromatin mediated by CHD1 and H3K9me3 specific demethylase JMJD2A. IL-10 protects changes within histone PTMs induced by TNF or LPS that affected H3K4me3-specific methyltransferase SETD1A and MLL1. Neutrophils previously exposed to inflammatory factors become unvulnerable to IL-10 because previous LPS stimulation interrupts TSS regions marked by H3K4me3 of CHD1 and JMJD2A genes. Therefore, LPS-activated neutrophils are disabled to induce CHD1/JMJD2A enzymes by IL-10, making this process irreversible. Because transcription of JMJD2A and CHD1 also depends on TSS positioning by H3K4me3, neutrophils before LPS stimulation become insensitive to IL-10.

**Conclusion:**

Neutrophils, once pre-activated by TNF or directly stimulated by LPS, become insensitive to the anti-inflammatory effects of IL-10, and *vice versa*; IL-10 protects neutrophils against these proinflammatory stimuli. This phenomenon is responsible for disturbing the natural process of resolving inflammation and tissue regeneration.

**Graphical Abstract:**

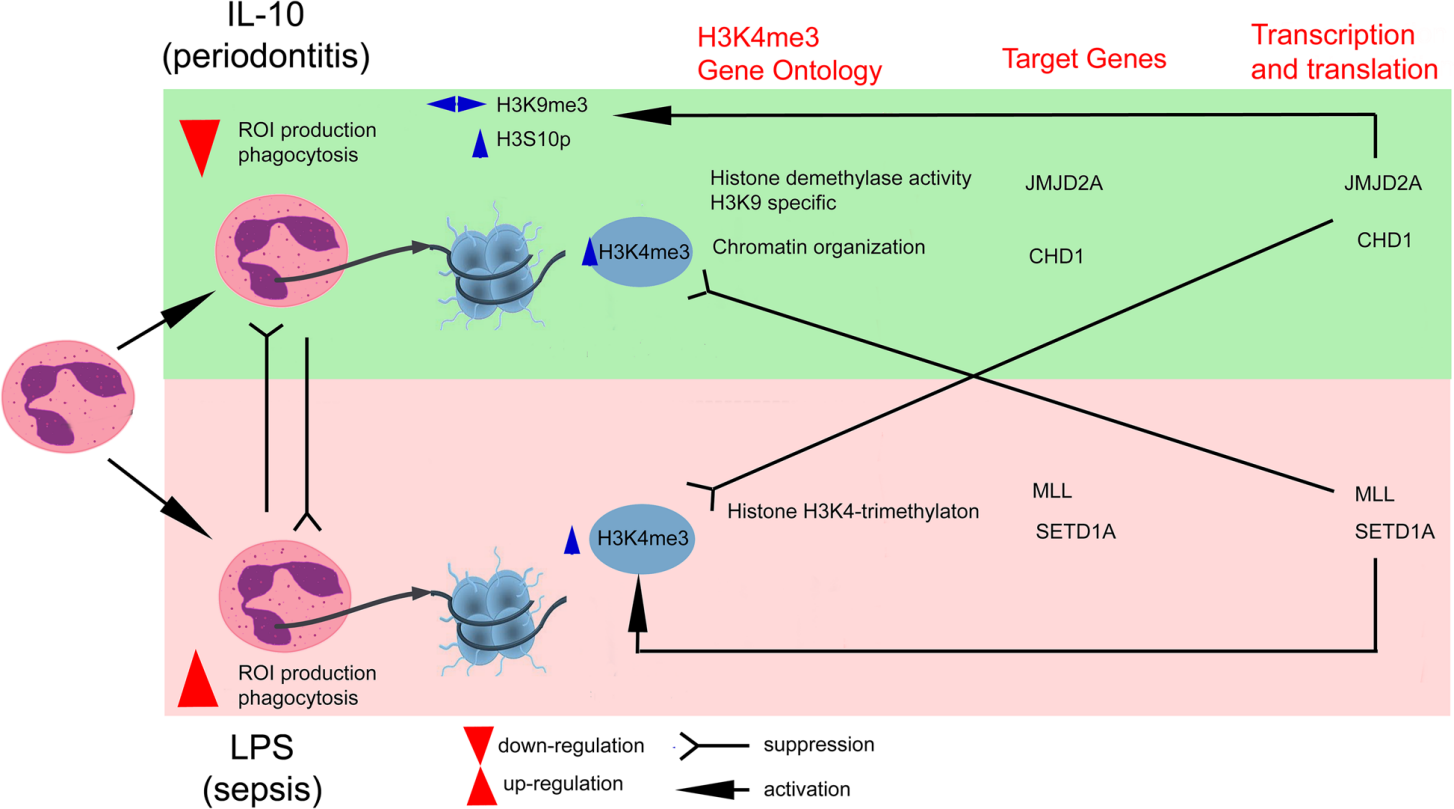

**Supplementary Information:**

The online version contains supplementary material available at 10.1186/s12950-024-00389-8.

## Highlight

PTM enzymes regulate neutrophil plasticity during different periods of inflammation. Neutrophils, once pre-activated by TNF or directly stimulated by LPS become insensitive to the anti-inflammatory effects of IL-10, and *vice versa*; IL-10 protects neutrophils against these proinflammatory stimuli. This phenomenon is responsible for disturbing the natural process of resolving inflammation and tissue regeneration.

**Supplementary Information:**

The online version contains supplementary material available at 10.1186/s12950-024-00389-8.

## Introduction

 Neutrophils, as the fastest-reacting cells in response to pathogens, with loosely arranged nuclear chromatin facilitating access of transcription factors to DNA in a relatively short time after stimulation, are supposed to be ‘ready’ for rapid initiation of gene expression related to immune response [1]. High antimicrobial potential acts as a double sword, as uncontrolled activation of neutrophils can damage surrounding tissues or lead to increased mortality because of cachexia during activation in the bloodstream [2–4]. For this reason, neutrophil physiological activation is under control of two steps, which prevent them from uncontrolled initiation beyond inflammatory sites and with adequate force to pathogens. The first step in blood vessels prepares neutrophils for a more effective response. It is initiated by a low concentration of proinflammatory cytokine/chemokine or components of the complement pathway by signals from the ongoing inflammation [5]. The second one is the direct response to adequately recognised pathogens *via* pathogen molecular pattern manner. Our previous studies also discovered other mechanisms regulating the immune response orchestrated by neutrophils [6]. This phenomenon, called ‘infection tolerance,’ is initiated by neutrophil-LPS-stimulated Treg interaction, which results in the repolarisation of neutrophils to IL-10-positive cells, which, in the second phase, induces other IL-10-secreting neutrophils in a paracrine manner. We postulate that this two-step reaction results in massive neutrophil apoptosis, occurring during resolving acute inflammation or replacing the innate immunity to acquire one. It is worth emphasising that this mechanism can occur without proinflammatory factors observed within prolonged chronic inflammation or direct (without process of pre-activation) LPS stimulation [7]. The process of silencing neutrophil antimicrobial properties is essential in tissue regeneration after infections, as prolonged inflammation leads to tissue damage [8]. Continuing our studies, we discovered that despite IL-10-induced apoptosis, it can also switch the profile of cytokines/chemokines/growth factors desired in resolving inflammation *via* non-canonical NF-kB pathway with simultaneous canonical NF-kB pathway inhibition [9]. IL-10-stimulated neutrophils are characterised by a significant secretion of vascular endothelial growth factor (VEGF) and CXC chemokines. VEGF performs its function by stimulating extracellular matrix remodelling, enhancing endothelial cell proliferation and migration, and increasing vascular permeability, resulting in fibrinogen leakage into the perivascular environment [10]. In turn, CXC chemokines (CXCL1, CXCL2, CXCL5, CXCL8, CXCL10, MIF CCL19) associated with induction of angiogenesis by ligation of the CXCR2 recruit and moderate the function of macrophages as well as dendritic cells [11]. It has been postulated that neutrophils are actively involved in resolving inflammation by transparent tissues from infiltrated leucocytes and creating a specific environment desired for tissue architecture and function repair [12, 13].

The described specific ‘polarisation’ of immune cells is mediated by the modification of chromatin, which enables differentiated access and activity of regulatory elements that guarantee their plasticity during inflammation [14]. The nucleosome consists of H2A, H2B, H3, and H4 histones susceptible to numerous post-translational modifications (PTMs) along their entire length. The correlation between certain histone modifications and specific gene expression led to the concept that they directly ‘instruct’ transcriptional activation or repression. The combinations of histone modifications are controlled by histone-modifying enzymes that determine diverse DNA scaffold processes, including gene expression [15].

Functional specialisation of the cell and epigenetic ‘code’ are closely linked. Cildir and colleagues demonstrated a positive correlation between histone H3 lysine K4 trimethylated (H3K4me3) and histone H3 lysine K27 acetylated (H3K27ac) in mast cells upon immunoglobulin E-mediated cross-linking of the IgEε receptors [16]. Gene transcription is controlled by enhancers that interplay with gene promotors as gene-distal regulatory elements, increasing gene transcription and characterised by the presence of H3K27ac histones [17]. Contrary to enhancers, transcriptional start sites (TSSs) are located mainly within H3K4me3 domains [18]. Our previous studies have revealed that circulating neutrophils of HIV-infected subjects compared to healthy controls are characterised by high levels of H3K4me3 histone and low H3Ac. Peripheral neutrophils in HIV-infected individuals are characterised by impairment of their antimicrobial properties, including the ability to chemotaxis, phagocytosis, and oxidative burst potential. ChiP-Seq H3K4me3, bioinformatic analysis of Gene Ontology, additionally revealed significant changes within target genes related to the cell activation, cytokine production, adhesive molecule expression, NF-kB transcription factor in canonical pathways, and also target genes within histone remodelling *via* upregulation of methyltransferase. Therefore, we conclude that the primary source of neutrophil dysfunction in HIV patients is interplayed between H3Ac and H3K4me3, which results in disturbing gene positioning associated with antimicrobial properties [19]. This data proposed that neutrophil pre-activation, which prepares neutrophils to respond more intensely to pathogens, can be related to H3K27ac, contrary to their direct stimulation mediated by H3K4me3 positioning.

In this study, we have focused on two processes: the physiological overriding mechanism, which reflects the shifting nature of neutrophils from cells ready to neutralise pathogens to cells reducing inflammation by initiating tissue regeneration after IL-10 exposure and the pathological mechanism disturbing this phenomenon by prolongated pre-activation observed in patients suffering from autoimmune disease and after direct activation by LPS observed during sepsis.

Using the in vitro model of inflammation, we have analysed 11 different histone PTMs (marked *inter alia*: active promotors, transcribed regions, repression genes, enhancer regions), PTM enzymes associated with remodelling neutrophil chromatin, and H3K4me3 ChIP-Seq Gene Ontology analysis focusing on the processes related to histone PTMs. Finally, this data was validated by comparison to adequate clinical status corresponding to the pathological neutrophil condition observed in Neuromyelitis Optica Spectrum Disorders (NMOSD) as an autoimmune disease, *E.coli*-induced sepsis vs. healthy volunteers. Periodontitis-derived circulating neutrophils were used as a physiological response of neutrophils to IL-10.

## Materials and methods

### Study design

In the first step of our investigation, using isolated circuiting neutrophils from healthy volunteers, we defined mechanisms involved in neutrophil TNF-pre-activation, LPS direct stimulation and IL-10-induced suppressor/tissue regeneration neutrophils. We made two assumptions: the first, that the antimicrobial and regenerative properties should be excluded as processes that do not co-occur; the second follows from the first, that the prior action of pro-inflammatory factors precludes the activity of IL-10 and vice versa – IL-10 prevents pre- and activation of neutrophils. To demonstrate this process, we stimulated neutrophils in a different order of TNF, LPS or IL-10. 2 × 10^6^ of neutrophils were incubated in vitro without stimulation, and in the presence of 100ng/ml ultrapure LPS from *E. coli* (serotype R515, Alexis Biochemicals), 100ng/ml human TNF (recombinant, expressed in *E. coli*; Sigma-Aldrich, St. Louis, MO, USA), and 100ng/ml human IL-10 (expressed in Sf21 insect cells, Sigma-Aldrich, St. Louis, MO, USA) in RPMI 1640 for 5 h (5% CO_2_, 37 °C, humid atmosphere). In the second step, we verified the thesis by extrapolating obtained data to an adequate clinical status where circulating neutrophils are permanently exposed to pro-inflammatory factors and characterised by prolongated life spine: neutrophils isolated from the blood of sepsis patients as LPS-stimulated [20], Neuromyelitis Optica Spectrum Disorders as an autoimmune disease with pre-activated neutrophils [21] as the control of physiological response- neutrophils vulnerable to IL-10, circuiting neutrophils from periodontitis patients were used [7]. Isolated neutrophils were incubated for 5 h in RPMI under similar conditions as neutrophils used in the in vitro model.

There were four independent replicates for each experiment in the in vitro model. To validate results from ChIP-Seq analysis obtained from the in vitro model, three representative patients with functional test parameters closest to the mean values of their group were classified to ChIP-Seq analysis.

### Assumptions for PTM selection

First, we selected modifications that seemed important in changing the nature of neutrophils from pro- to anti-inflammatory cells after IL-10 stimulation in a relatively short time (10 min). Therefore, we focused on PTMs marked active promotors and transcribed regions, assuming that neutrophils must be ready to differentiate the pro- vs. anti-inflammatory signal by triggering genes that engage chromatin-modifying enzymes. This process can be mediated by positioning the TSS region (H3K4me3) and active promotors (H3K36me3, H3K79me2) of transcribed regions. Simultaneously, we suspect that modification of transcription suppressor occurs to hide pro-inflammatory genes (H3K9me3, H3K27me3) and PTMs responsible for initiating genes within heterochromatin (H3S10p). Second, we selected modifications enhancing antimicrobial neutrophil properties observed during neutrophil TNF-preactivation. We suspect such neutrophils’ properties must be mediated by H3K27ac – PTM, described as a transcription enhancer. Third, as neutrophils leaving the bone marrow are already prepared to neutralise pathogens, we assume that LPS stimulation directly positions TSS regions by H3K4me3.

### Patients

#### Neuromyelitis Optica Spectrum Disorders (NMOSD)

Three patients with confirmed AQP4-IgG seropositive NMOSD, fulfilling the 2015 Wingerchuk criteria, were selected for ChIP-Seq analyses [22]. All the patients were without systemic steroids or other anti-inflammatory drugs for at least three months before the study.

#### Sepsis

Six patients with sepsis developed at least two symptoms from the following criteria: body temperature above 38℃ or below 36℃; pulse rate > 90/min; respiratory rate > 20/min or PaCO_2_ < 32 mmHg; leukocyte > 12,000/µL or < 4000/µL were classified for investigations [23]. Patients who had not received antibiotics for at least three months before the study and with positive microbial tests for *E. coli* in the blood were selected for ChIP-Seq analyses.

#### Periodontitis

Twelve patients with generalised stage III and IV periodontitis were selected for this study. The criteria of the Classification of Periodontal and Peri-Implant Diseases and Conditions 2017 [24] were used for periodontitis staging and classified for further investigation. Patients who had not received antibiotics or anti-inflammatory drugs for at least three months before the study were selected for ChIP-Seq analyses.

All participants of the study were diagnosed and recruited at the Department of Neurology (patients with NMOSD), at the I Department of Urology (patients with sepsis) and at the Department of Periodontology and Oral Mucosal Diseases (patients with periodontitis and healthy controls), all from Medical University of Lodz.

### Neutrophil isolation

20 mL of the whole blood on lithium heparin anticoagulant was collected from sepsis, NMOSD, periodontitis patients and HC. Neutrophils were purified by negative selection by microbeads, which allowed the removal of DCs, B cells, monocytes, macrophages, activated T cells, and activated NK cells (MACSxpress Whole Blood Neutrophil Isolation Kit, Miltenyi Biotec GmbH, Germany). Residual erythrocytes were lysed using 2mL ammonium chloride Lysing Reagent (BD Biosciences) for 5 min. The final purity of the neutrophil population was assessed by flow cytometry using CD14-PE (clone M5E2), CD15-FITC (MMA), and CD16-PECy7 (3G8, all from BD Pharmingen) mAbs. Flow cytometric analysis of the isolated cell population showed that the percentage of CD15^high^CD16^+^CD14^−^ neutrophils was > 98%. The level of contaminating CD14^+^CD15^+^ monocytes was about 0.4%, and CD15^+^CD16^−^ eosinophils was < 0.1% after isolation, as we proved in our previous studies [7, 9, 19].

### Phagocytosis and reactive oxygen intermediates (ROI) production

With some modification, ready-to-use kits were used to analyse neutrophil phagocytosis and ROI production (Bursttest and Phagoburst, OrphoGen Pharma, Heidelberg, Germany). Briefly, isolated neutrophils were stimulated by different LPS, TNF and IL-10 constellations. Each stimulator was added to a 500 mL suspended cell for 10 min, 37 °C, in a water bath. The total time of neutrophil stimulation in this part of the experiment was 20 min. After incubation, samples were cooled down in an ice bath for 10 min. The ability of neutrophils to phagocytosis was performed on *E. coli* conjugated with FITC (3.3 × 10^8^ bacteria/mL) and presented as the mean fluorescence intensity acquired from intercellular FITC signal. The ROI production was performed with or without restimulation by PMA (phorbol 12-myristate 13-acetate, 0.162 µM, 10 min) and presented as the mean intensity of 1,2,3-dihydrorhodamine (1,2,3-DHR), whose reduction depends on intracellular ROI concentration. Samples were analysed within 30 min using flow cytometry (BD LSRII, FACSDiva™).

### Analysis of cell surface markers

The surface expression of CD11b, CD18 and CD62L were determined using BD LSRII flow cytometry and FACSDiva™ analysis software. After incubation with different stimuli combinations, the samples were washed, labelled with mAbs, fixed with 1% paraformaldehyde and analysed. For immunostaining, conjugated CD11b-PE (clone D12, BD Biosciences, CA, USA) CD18-FITC (clone 6.7, BD Biosciences, CA, USA), CD62L-FITC (clone SK11, BD Biosciences, CA, USA), antibodies and respective mouse isotype controls: IgG2a-PE (DAK-G05, Daco A/S, Denmark) and IgG1-FITC (DAK-G01, Daco A/S, Denmark) were employed.

### Levels of post-translation histone modification by dot blot analysis

The stimulation of neutrophils (2 mln cells resuspended in 2mL RPMI) by different LPS, TNF and IL-10 constellations was stopped by ice-cold PBS rinsing. Subsequently, neutrophils were lysed on ice in Pierce™ RIPA buffer (ThermoScientific) containing Halt™ Protease & Phosphatase Inhibitor Cocktail (ThermoScientific) for 5 min and sonicated using Bioruptor® Pico Sonicator (Diagenode, five cycles; 30 s. “ON” 30 s. “OFF”). The lysates were collected and centrifuged at 12,000×*g* for 10 min at 4 °C. The protein concentrations were determined by a Nanodrop (ThermoFisher). The protein samples were transferred to 0.2 μm nitrocellulose membrane (Bio-Rad) for immunoblotting (20 µg to each reaction). The membrane was incubated for 50 min in RT with primary antibodies: anti-monomethyl-Histone H3 Lys4 (H3K4me1, rabbit, 1:100), anti-dimethyl-Histone H3 Lys79 (H3K79me2; clone NL59, 1:500), anti-phospho-Histone H3 Ser10 (H3S10p, clone MC463, 1:500), anti-trimethyl-Histone H3 Lys36 (H3K36me3, rabbit, 1:500), anti-trimethyl-Histone H3 Lys27 (H3K27me3, rabbit, 1:1000), anti-acetyl-Histone H4 Lys16 (H4K16ac, rabbit, 1:5000), anti-acetyl-Histone H3 Lys27 (H3K27ac, rabbit, 1:2000), anti-trimethyl-Histone H3 Lys4 (H3K4me3, rabbit, 1:500), anti-acetyl-Histone H3 Lys9 (H3K9ac, rabbit, 1:1000), anti-phospho-Histone H2 Thr120 (H2Ap, rabbit, 1:1000), anti-trimethyl-Histone H3 Lys9 (H3K9me3, rabbit, 1:500) and GAPDG (1:5000) as an endogenous control. All antibodies dedicated to ChIP-Seq analysis were obtained from Merck-Millipore. Subsequently, the membrane was ‘blocked’ by SuperBlock™ Blocking Buffer- Blotting in TBS. After 1 h of incubation, the RT membrane was 3x washed by TTBS buffer and secondary anti-rabbit IgG peroxidase conjugate antibodies were added for 35 min at RT (1:3000, Sigma Aldrich. The membrane was washed 2x by TTBS and 2x by TBS. Dot-blotting was performed using the ECL chemiluminescence peroxide solution (Bio-Rad). The densitometric scanning of the dot blots and analysis of the visualised spots was performed using a G-Box (Syngene) and Genesys Image Acquisition software (version 1.2.5.0). The results are presented as the intensity of optical density of the area underspot’spots’ peak (average value of the pixels enclosed after background correction) normalised to GAPDH.

### Immunocytochemical analysis (ICC)

ICC analysis was performed on isolated neutrophils stimulated by LPS, TNF or IL-10 in different sequences (in vitro model) and on neutrophils incubated in RPMI and obtained from patients: NMOSD, sepsis or periodontitis (neutrophils stimulated in vivo). Neutrophils were transferred to poly-L-lysine-coated microscope slides by cytospin (300xg,10 min) and fixed with 4% formaldehyde solution for 6 min at RT. Fixed cells were washed with PBS and blocked with 10% rabbit blocking serum (Santa Cruz Biotechnology, Dallas, TX, USA) supplemented with 0.3% Triton™ X-100 (Sigma-Aldrich, St. Louis, MO, USA) for 45 min at 21℃. Next, they were washed and double-stained for SETD1A/MLL1, JMJD2A/CHD1, H3S10p, and H3K27ac. Anti-SETD1A (2 µg/ml, mouse, Proteintech, Manchester, UK), anti-MLL1 (KMT2A, 2 µg/ml, rabbit, Proteintech, Manchester, UK), anti-JMJD2 (1:100, D-9, mouse, Santa Cruz Biotechnology, Dallas, TX, USA), anti-CHD1 (2 µg/ml, mouse, Proteintech, Manchester, UK), anti-H3S10p (rabbit, clone MC463, 1:300, Merck-Millipore, Temecula, USA), anti-H3K27ac (rabbit, clone Lys27, 1:300, Merck-Millipore, Temecula, USA) and human IgG Isotype Control (Invitrogen, Cat.#31,154) as negative primary antibody control, were used. All antibodies were suspended in PBS supplemented with 1.5% blocking rabbit serum, 0.3% Triton X-100, and 0.01% sodium aide and incubated overnight at 4 °C. Cells were washed, and secondary fluorescent Abs were added one or 1 h at RT: goat pAb to mouse TR (5 µg/ml, cat. T862, Invitrogen, USA) with goat pAbs to rabbit FITC (2 µg/ml, cat. F2765, Invitrogen, USA). As isotype secondary antibody controls, goat IgG F(ab’)2 FITC (Invitrogen, Cat. #11,301 C) and rat IgG2a Texas Red (Invitrogen, cat# R2A17) were used. DAPI (1.5 µg/ml UltraCruz Mounting Medium, Santa Cruz Biotechnology, Dallas, TX, USA) was used for nuclei DNA staining. The confocal laser scanning microscopy platform TCS SP8 (Leica Microsystems, Germany) with the objective 63×/1.40 (HC PL APO CS2, Leica Microsystems, Germany) was used for microscopic imaging. Leica Application Suite X (LAS X, Leica Microsystems, Germany) was used for cell imaging. Fluorescence intensity was determined in the Region of Interest as the sum of the fluorescence from all segments (bordered by the line) divided by their number (arbitrary units- a.u.). The average fluorescence was calculated using at least 100 single cells for each sample. Nonspecific fluorescence (signal noise) was electronically diminished to the level when the nonspecific signal was undetectable (background). ICC data were additionally presented as the values of Colocalization Rate that indicate the overlap of the fluorescence signals between the channels FITC, TR and DAPI (nucleus). It was calculated as the mean value of a single Region of Interest using Leica Microsystem (LAS-X, ver. 3.7.020979 software, Leica, Germany).

### H3K4me3 ChIP-Seq analysis

#### Chromatin immunoprecipitation (ChIP)

Neutrophils stimulated by LPS, TNF or IL-10 (in vitro model) and isolated from patients: NMOSD, sepsis, and periodontitis (neutrophils stimulated in vivo) were used to analyse DNA coupled with H3K4me3 histone. ChIP was carried out in neutrophils according to the manual of Magna ChIP™ A/G Chromatin Immunoprecipitation Kit (Merck Millipore). Cells were fixed with 1% formaldehyde in RPMI solution for 10 min at RT and quenched with 10x glycine in 5-minute incubation at RT to stop the fixation. After washing with cold PBS, cells were treated sequentially with 1x Protease Inhibitor Cocktail II, Lysis Buffer with Protease Inhibitor Cocktail II, and Protease Inhibitor Cocktail II with Nuclear Lysis Buffer. The supernatant was removed, and the cell pellet was resuspended in a Nuclear Lysis Buffer. Sonication (10 cycles; 30 s. “ON” 30 s. “OFF”) was done using Bioruptor® Pico Sonicator (Diagenode, Belgium). The obtained chromatin was spun at a minimum of 10,000 x g at 4 °C for 10 min to remove insoluble material. Each immunoprecipitation required the addition of Dilution Buffer and Protease Inhibitor Cocktail II. 25 µL of the diluted chromatin as ‘Input’ was saved at 4 °C for further proceeding. Chromatin immunoprecipitation was performed with the use of the set of antibodies: Normal mouse IgG (negative control), anti-RNA Polymerase II (clone CTD4H8) as positive control and anti-trimethyl-Histone H3 (Lys4) (MC315, Merck Millipore) mAbs. Both antibodies were recommended for use in the ChIP-Seq technique [25]. Immunoprecipitation reactions were incubated overnight at 4 °C with rotation. DNA was eluted and purified using spin columns. The DNA concentrations of obtained samples were measured by Qubit 4 Fluorometer (ThermoFisher Scientific).

#### Library preparation and NGS sequencing

Double-stranded DNA was generated from a single-stranded fraction of ChIPed DNA using NEBNext® Ultra™ II Non-Directional RNA Second Strand Synthesis Module (E6111S, New England Biolabs). The reaction was performed with random primers from NEBNext® RNA First Strand Synthesis Module (E7525, New England Biolabs). Libraries for sequencing were prepared using NEBNext® Ultra™ II DNA Library Prep Kit for Illumina® (E7645L, New England Biolabs). Single-end sequencing with a read length of 75 bases (SE75) was performed with NextSeq550 (Illumina) to obtain at least 20 million reads per sample that could be mapped to the human genome [26].

#### Bioinformatic methodology of the ChIP-Seq analysis

In the first stage, the quality of the raw sequence reads was checked using the FASTQC software (version: 0.11.8). Next, all reads were subjected to the adapter, and quality filtering (minimum quality (-q 25), minimum length (-m 15) using the Cutadapt tool (version: 1.18) in NextSeq reads mode. Trimmed reads were aligned to the reference genome (GRCh38) using the Bowtie2 (version: 2.2.9) in the single-end mode. Duplicated reads were located and tagged using the Picard MarkDuplicates tool (version: 2.18.4). Reads with low mapping quality score (MAPQ < 10) were removed from downstream analysis with the Samtools software (version: 1.6). Protein binding sites identification in the previously prepared BAM files was performed with the MACS2 (Model-based Analysis of ChIP-Seq) software (version: 2.1.0) in narrow peak mode [27]. Subsequently, identified peaks were annotated using annotatePeaks.pl from Homer software (version: 4.11.1, hg38 annotation library). Functional enrichment analysis for various categories (molecular function, biological process, cellular component and pathways interaction) was also executed [28]. To find enriched motifs in ChIP-Seq peaks, the findMotifsGenome.pl program from Homer software (version: 4.11.1) was used. The quantitative assessment of ChIP-Seq quality was checked by applying the ChIPQC package (version: 1.21.0) from R Bioconductor (version: 3.6.0). Differentially enriched sites between experimental conditions were identified using the DiffBind package (version: 2.12.0) from R Bioconductor (version: 3.6.0).

ChIP-Seq library quality control analysis and assessment of ChIP-Seq quality were provided in our previous studies [9].

### mRNA analysis of SETD1, MLL, CHD1 and JMJD2A by real-time quantitative PCR

Total RNA was extracted from neutrophils with a mirVana™ miRNA Kit (Thermo Fisher Scientific). An Agilent small RNA kit was used to estimate purity and total RNA concentration (2100 Bioanalyzer, Agilent 2100 expert software). RNA was transcribed into cDNA using iScript Reverse Transcription Supermix (BioRad, USA). cDNA was amplified in the presence of TaqMan specific primers: JMJD2A (F 5’TGCGGCAAGTTGAGGATGGTCT3’; R 5’GCTGCTTGTTCTTCCTCCTCATC3’), CHD1 (F 5’TCTCTTCCTGCCAAGGTTGAGC3’; R 5’TGCCCTTGGAACCTTTGCTGAG3’),

MLL(KMT2A) (F 5’GTGCTTTGTGGTCAGCGGAAGT3’;

R 5’TGTGAGACAGCAACCCACGGTG3’),

SETD1A (F 5’TCGAGAGGAAGCTGTGGATACC3’; R 5’CGCCATCTGAGTCAGCATACAG3’) using 7500 Real-Time PCR System (Applied Biosystems) according to the following program: 95 °C 10 min; 40 cycles of (95 °C, 15s; 60 °C, 60s). We used RT2 Real-Time™ SYBR Green/PCR Master Mix (Qiagen, UK) that contains all reagents and buffers required for qRT-PCR. The expression levels of the β-actin were used for the normalisation of the cDNA samples (Ct value). The relative expression to non-stimulated neutrophils was calculated using the ΔΔCt method.

### Statistics

Arithmetic means and standard deviations were calculated for all parameters. A statistical analysis of differences was performed using the one-way ANOVA (comparison between ‘n.s.’, LPS- and TNF-stimulated neutrophils) or the two-way ANOVA test (analysis of IL-10 on LPS stimulation or TNF-preactivation processes). Tukey’s test was used for multiple comparisons as a *post hoc* test when statistical significance was identified in the ANOVA test. The statistical comparison between clinical groups (sepsis, NMOSD and periodontitis patients) and healthy volunteers was performed using the Student-t test. *p* ≤ 0.05 was considered as the significant difference.

## Results

### IL-10 protects neutrophils against pro-inflammatory stimuli

**Fig. 1:**
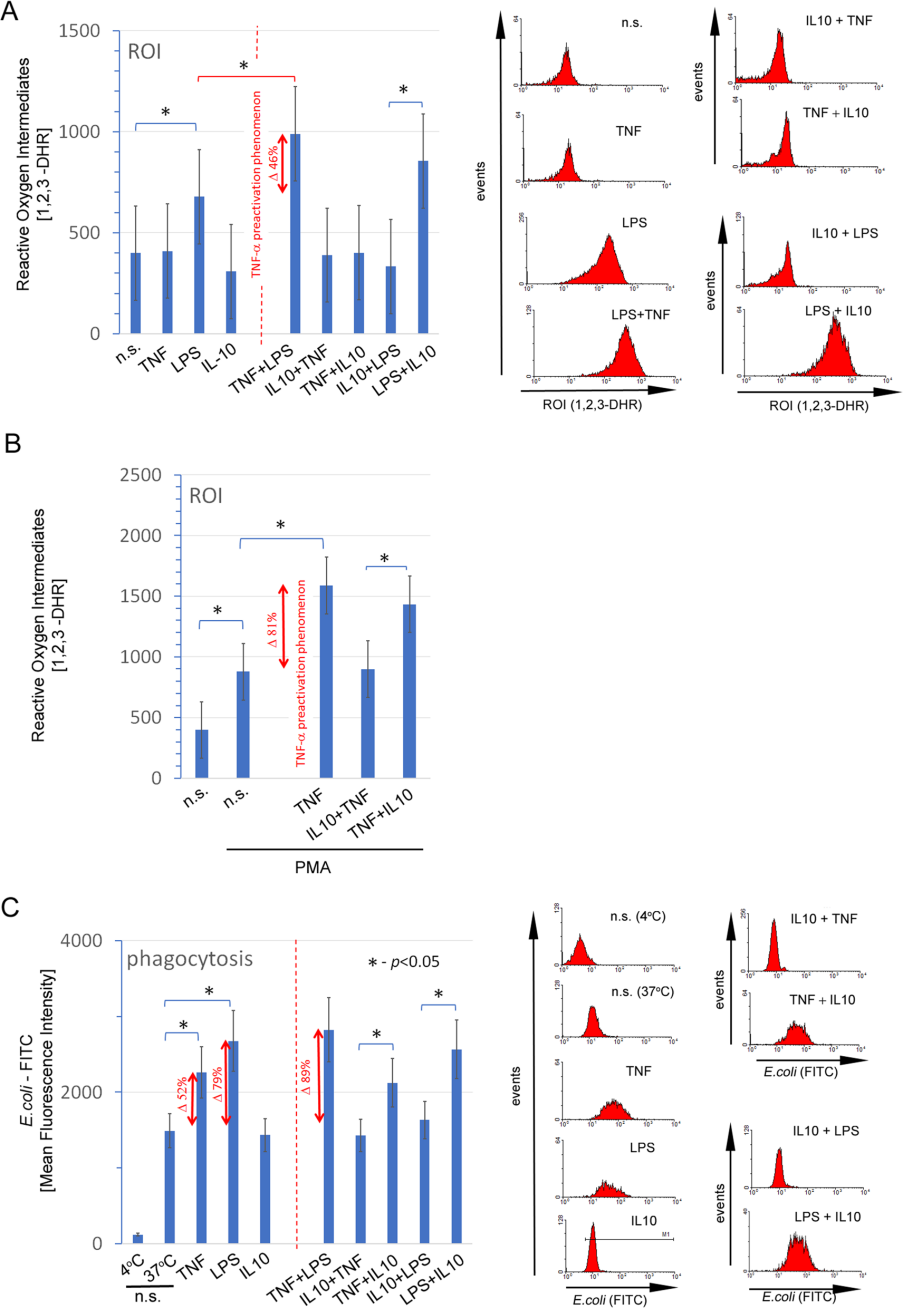
IL-10 protects neutrophils against pre- and activation provided they had not been previously exposed to pro-inflammatory agents such as TNF or LPS. **A** The direct exposition of TNF, LPS, IL-10 on ROI production and the effect of IL-10 on the neutrophil pre- and activation process. The double-headed red arrow demonstrates the pre-activation phenomenon by TNF amplifying antimicrobial neutrophil properties. The low panel presents one of four independently performed experiments. **B** To better demonstrate the protective effect of IL-10 on the pre-activation, neutrophils were additionally stimulated by PMA, which directly activated NADPH oxidase. **C** The exposition of TNF, LPS, IL-10 on *E. coli* phagocytosis and the effect IL-10 on TNF-preactivated and LPS-stimulated neutrophils. The double-headed red arrow demonstrates the influence of TNF, LPS, IL-10 and TNF + LPS on neutrophil *E. coli* phagocytosis (left part of the graph). The right part of the graph (demarcated by a dashed line) demonstrates the protective effect of IL-10 on TNF-pre-activated and LPS-activated neutrophils and the disruption of this process due to previous short-term exposure to TNF or LPS. The rights panels present the most representative example of four independent experiments

In the first set of experiments, we analysed the influence of IL-10 on two main processes: reactive oxygen production after LPS stimulation and the ability of *E. coli* to be phagocytosed. To reflect the natural process at different times of inflammation, we assumed that neutrophils are susceptible to pro-inflammatory factors in the early period of inflammation, but in the final period, they should be sensitive to anti-inflammatory IL-10. First, we have demonstrated the phenomenon of pre-activation. Neutrophils previously exposed to TNF for 10 min were characterised by significantly greater ROI production after 10 min of LPS or PMA stimulation (Fig. 1A, B) and more efficient *E. Coli* phagocytosis process vs. non-preactivated cells (Fig. 1C).

The pre-activation phenomenon is spectacularly demonstrated with the ROI production after PMA stimulation (81% increase in neutrophils previously exposed to TNF (Figure 1B). This effect is not simply additive, as TNF-acting alone did not stimulate ROI production. Surprisingly, we didn’t observe the preactivation phenomenon during phagocytosis as no differences have been observed between LPS acting alone vs. TNF + LPS (Fig. 1C). It is worth adding that in our previous investigation, we noted a slight, but statistically significant, increase in the ROI production after TNF-preactivation [9]. This discrepancy is the result of different ways of interrupting the TNF activation. In previous manuscripts, we used only one of each factor for 10 min and after that samples were washed with ice-cold RPMI to stop the reaction and remove stimuli. In this experiment, TNF-preactivated neutrophils had to wait for 10–12 min. on the ice for the rest of the samples: TNF + IL10, IL10 + TNF and then were rinsed with ice-cold RPMI.

Next, we have focused on the CD11b/CD18 heterodimers (MAC-1) as the most prevalent member of β2 integrin specifically expressed on neutrophils [29]. Both, adhesion molecules CD11b and CD18, increased their expression within 10 min of exposition on TNF, LPS or IL-10. The most spectacular increase has been observed during TNF exposition and subsequent in stimulation with LPS. This effect can be additive as both stimuli increased CD11b as well as CD18 molecules to a similar degree (Supplementary Fig. 1A, B).

Following, we have focused on the effectiveness of IL-10 in preventing neutrophils from pre- and activation by proinflammatory stimuli estimated by the ability to ROI production, phagocytosis as well as CD11b/CD18 molecule adhesion expression. We noted that 10 min of IL-10 neutrophil stimulation made them insensitive to TNF-preactivation or direct LPS stimulation. This phenomenon is observed in all estimating parameters. IL-10 had no such properties if neutrophils were previously exposed to TNF or LPS. In Fig. 1 the right part of each graph presents ROI production and phagocytosis, while in Supplementary Fig. 1A and, B right panels of each graph and low panels demonstrated the effect of IL-10 on CD11b/CD18 expression.

Another important molecule responsible for adhesion, transendothelial migration (TAM, diapedesis) and neutrophil preactivation is L selectin (CD62L). Loss of CD62L expression is used as a gold standard to assess neutrophil activation which can correspond with increased CD11b/CD18 expression [30]. As expected, a 10-minute exposition of TNF and LPS decreased CD62L on the surface of neutrophil. Unlike CD11b/CD18 expression, we did not observe the effects of IL-10 on shedding CD62L (Supplementary Fig. 1C upper and middle panels). Next, we have focused on the effectiveness of IL-10 in preventing the shedding of CD62L molecules induced during TNF preactivation and LPS activation. We noted that IL-10 stimulation made neutrophils insensitive to the shedding of CD62L by TNF or LPS stimulation. Similar to the tests described above, IL-10 had no such properties if neutrophils were previously exposed to TNF or LPS (Supplementary Fig. 1C low and upper panels).

These experiments elucidate why neutrophils, in acute and chronic inflammatory conditions, become insensitive to IL-10 until the inflammatory agents are removed. In both sepsis induced by Gram-negative bacteria and autoimmune diseases such as NMOSD, circulating neutrophils are persistently activated or preactivated and are not susceptible to the anti-inflammatory effects of IL-10 even though high concentrations are observed in serum (sepsis, not surviving patients) or in serum and cerebrospinal fluid in NMOSD [31, 32].

We speculate that such crucial changes in neutrophil function must occur at the genomic level by blocking and/or initiating more transcription genes. Therefore, we analysed 11 histone PTMs that characterised hetero- and/or euchromatin.

### Il-10 protects neutrophils from being polarised into pro-inflammatory cells by altering PTMs

**Fig. 2:**
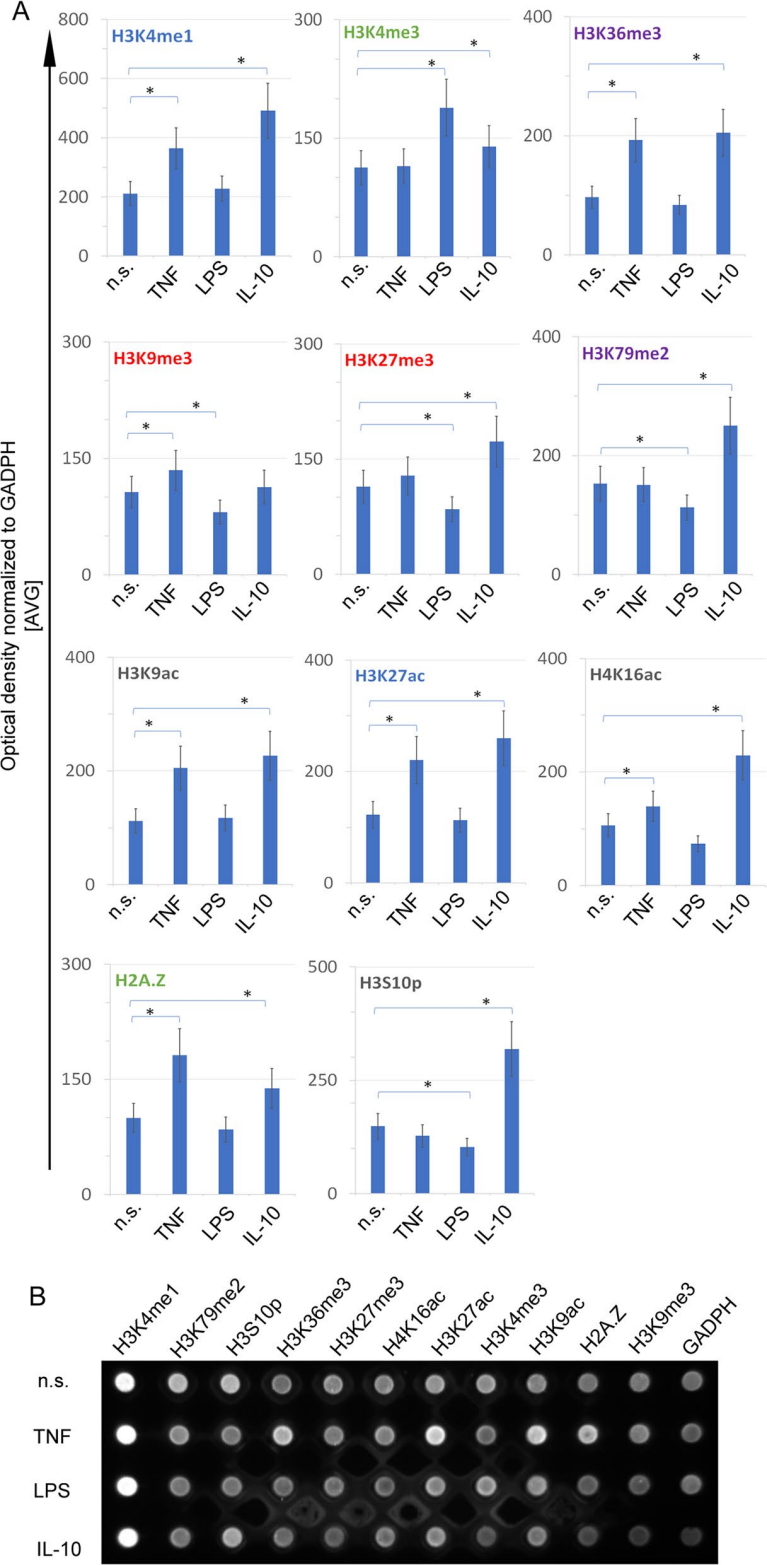
Patterns of selected posttranslational histone modifications characterised non-stimulated, TNF-preactivated, LPS- and IL-10-activated neutrophils. **A** Average level ± SD of different posttranslational histone modifications performed on four independent experiments. Statistical significance was compared to ‘n.s.’ neutrophils; active promotors are signed in green, transcribed region in purple, repression genes in red, and enhancer regions in blue colour. **B** The example of dot blot analysis

 First, we have discovered that neutrophils show different PTM profiles depending on their pre-activated, activated or immunosuppressed state. TNF-preactivated neutrophils are characterized by high level of H3K36me3, H3K79me3, H3K4me1, H3K9ac, H3K27ac, H4K16ac and H2Ap, which mainly marked active transcribed regions (*inter alia* H3K36me3, H3K79me3) and enhancers (H3K4me1 and H3K27ac). In turn, LPS-activated neutrophils are specifically characterised by high levels of H3K4me3, which marked TSS regions and low H3K9me3, H3K79me2 and H3K27me3, which, in turn, marked repressed genes. IL-10 positively induced the level of H3K4me1, H3K4me3, H3K36me1, H3K27me3, H23K79me2, H3K9ac, H3K27ac, H4K16ac, H2Ap and H3S10p, which distinctly characterised active promotors, transcribed regions and enhancers (Fig. 2).

The PTM analysis of neutrophils pre-activated and subsequently activated by LPS revealed high levels of H3K4me1, H3K79me2, H3K36me3, H3K27me3, H4K16ac, H3K27ac, H3K4me3, H3K9Ac, H2Ap, H3K9me3 and diminished H3S10p (Supplementary Fig. 2, left part of each graph). Subsequently, we have analysed the effect of IL-10 before and after exposition to TNF or LPS. IL-10 added before pre-activation by TNF reduced H3K27me3 H3K9me3 and increased H3K27ac and H3S10p compared to the experiment with TNF added for 10 min before IL-10. IL-10 also prevented direct LPS activation of neutrophils by decreasing levels of H3K4me1, H3K4me3, H3K79me2, H3K36me3, H3K27me3, H3K27ac, H3K9Ac, H2Ap, and H3S10p compared to experiment with LPS added 10 min before IL-10 (Supplementary Fig. 2, right part of each graph).

### LPS-modified neutrophil histone H3K4 *via* activation of lysine methyltransferase SETD1A and MLL1

**Fig. 3:**
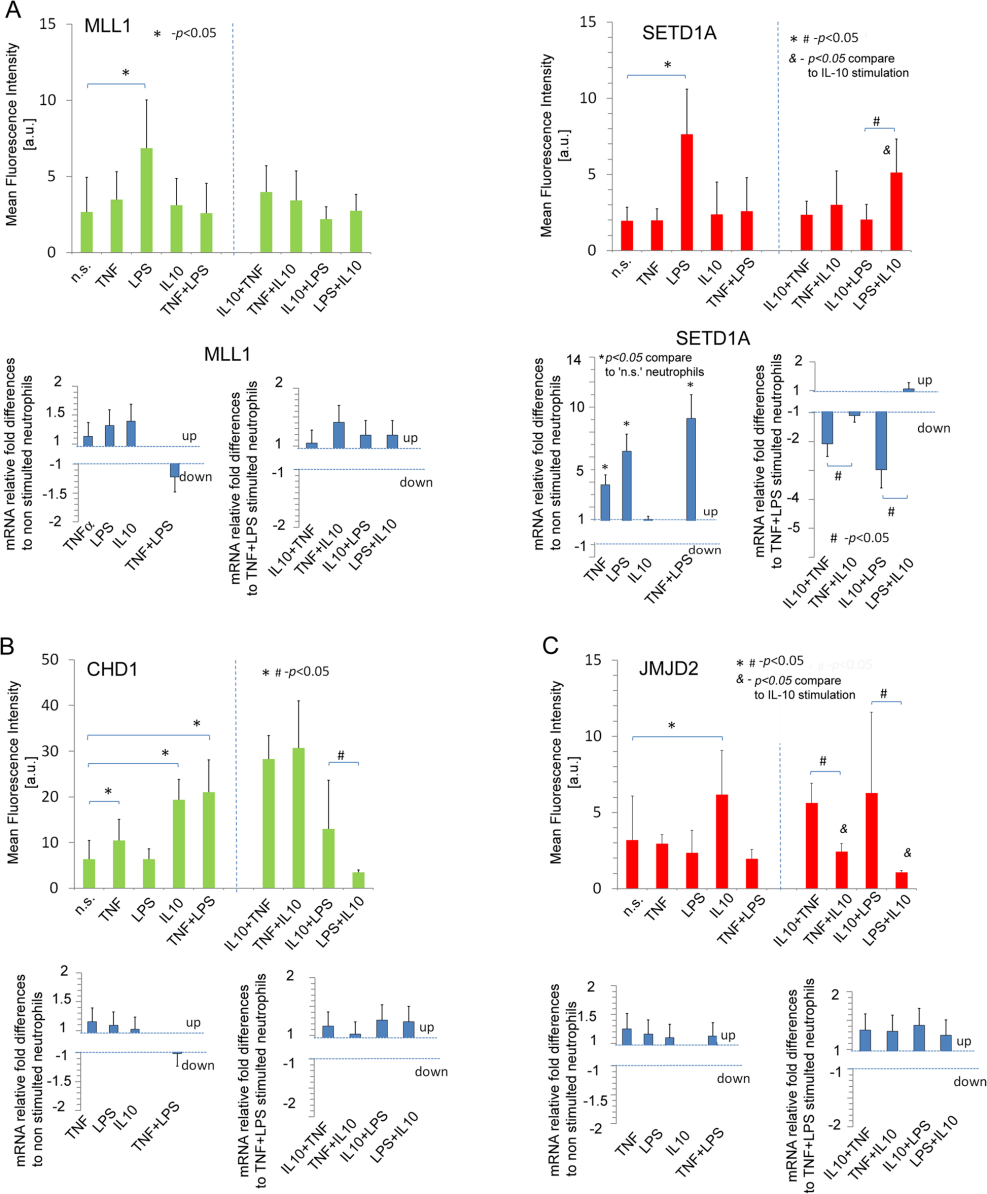
IL-10 prevents LPS direct stimulation and TNF-preactivation of neutrophils by regulating PTM-modified enzymes. **A** Increases in expression of MLL1 and SETD1 proteins, being the result of direct LPS effect (left part of the graph), are neutralised by IL-10 (right part of each graph). The protective effect of IL-10 occurs only when IL-10 first acts on neutrophils. The upper panels presented average protein levels and low panel mRNA expression ± SD. * - statistically significant differences within: n.s.; TNF, LPS, IL-10 or TNF + LPS samples. ^#^ - statistically significant differences within IL-10 + TNF, TNF + 10, IL-10 + LPS or LPS + IL-10. A separate statistical comparison within protein levels is marked with a dashed line. **B** CHD1 is overexpressed during direct exposure of neutrophils to IL-10 or TNF, but not LPS. Co-stimulation of neutrophils with IL-10 and LPS disturbs CHD1 expression regardless of the order of LPS administration. **C** IL-10, opposite to LPS or TNF exposition, triggered JMJD2A

As we have shown above, LPS-stimulated neutrophils were characterised, among others, by high H3K4me3 levels, which, in turn, were diminished by previous exposure to IL-10. This phenomenon suggests a prevailing role for H3K4me3 in IL-10 protective effect. Therefore, we have focused on the enzymes referred to as H3K4 ‘writers’ [33]. Histone H3 lysine can be modified by one, two or three methyl groups, and its levels and distribution reflect the combined action of methyltransferases and demethylases. In humans, H3K4 is methylated by the lysine methyltransferase SETD1A/B and MLL1-4 [34]. Using ICC methods, we have noted the high expression of SETD1A and MLL1 proteins after LPS but not TNF or IL-10 exposition (Fig. 3A, upper panel of each graph). The discrepancy between the outcomes of transcription and translation probably results from the different dynamics of both processes. TNF-preactivation which is characterized by intensified SETD1A translation, but not their transcription, is physiologically justified as the process associated with preparing neutrophils for anti-pathogen response. In turn, the direct exposition of neutrophils to LPS results in the intensification of both processes simultaneously.

The analysis of the colocalisation rate confirmed the appearance of these enzymes in the nucleus (Supplementary Fig. 3). The transcription analysis of these enzymes revealed a statistically significant increase in mRNA expression of SETD1A but not MLL1 after exposition on TNF or LPS to ‘n.s.’ neutrophils (Fig. 3A, upper panel). This set of experiments suggested that the induction mechanism of H3K4me3 PTMs by SETD1A depends on the positioning itself *via* a positive feedback mechanism contrary to MLL1, whose level is predefined and does not require transcription. The experiment with IL-10 exposition revealed that this cytokine prevents neutrophils from high expression of SETD1A and MLL1 resulting from LPS stimulation. IL-10 diminished SETD1A and MLL1 protein expression but only mRNA for SETD1A. The protective effect of IL-10 occurs only if IL-10 first acts on neutrophils before LPS or TNF-stimulation (Fig. 3A, upper right panels). This data suggests a mechanism of IL-10 preventing LPS stimulation mainly dependent on the SETD1A gene positioning and acting only when removing pro-inflammatory stimuli.

### IL-10 rearranges chromatin by CHD1 and JMJD2A induction

The crucial aspect of the overriding mechanism reflects the changing nature of neutrophils from cells ready to neutralise pathogens into cells that reduce inflammation and start tissue regeneration after IL-10 exposition. We speculated that such changes in neutrophil function, through the activation of initially inactive genes, should result from changes at the histone rearrangement level that engage chromatin remodelling enzymes triggering genes within heterochromatin. We have selected two enzymes that we thought to be involved in neutrophil polarity reversal: chromodomain helicase DNA-binding protein 1 (CHD1) and histone demethylase (JMJD2A/KDM4A). CHD1 cooperates with H3K4me3 as most likely associated with the rapid activation and positioning of transcription genes; in turn, JMJD2A, an enzyme that demethylates H3K9me3 positing genes within heterochromatin [35, 36]. The ICC analysis revealed high expression of CHD1 in neutrophils exposed to IL-10 during pre-activation by TNF as well as pre-activation and subsequent activation by LPS. It is noteworthy that prior LPS stimulation results in a blockade of CHD1 expression under the influence of IL-10. We have not observed any changes within CHD1 mRNA expression in any of the experimental setups (Fig. 3B). In the same experiment series, we noted high expression of JMJD2A in protein and mRNA levels in neutrophils exposed to IL-10. ICC analysis revealed increased expression of JMJD2A in the neutrophil nucleus exposed to IL-10 (Fig. 3C). The analysis of TNF or LPS influence on this process revealed that only neutrophils exposed first to IL-10 characterised with JMJD2A high expression, while additional exposure to LPS or TNF did not affect JMJD2A expression. In turn, neutrophils previously pre-activated by TNF or stimulated by LPS abort the influence of IL-10 on JMJD2A expression. A similar relationship was observed at the mRNA level, suggesting that induction of JMJD2A by IL-10 and blocking this process by TNF or LPS occur at the transcription level (Fig. 3C). The colocalisation of CHD1 and JMJD2A within DNA using z stock-confocal analysis has confirmed a high colocalisation rate in IL-10-stimulated neutrophils (Supplementary Fig. 4). It is worth noting that the location of CHD1 is on the periphery opposite to JMJD2 which is localised mainly in the centre of nuclei. This remark suggested acting these enzymes within different A/B segments of chromatin. Compartment A is active and located in the interior nuclear space, whereas compartment B is inactive and located in the nuclear lamina [37]. The high expression of CHD1 within compartment B confirms our assumption that this enzyme is involved in triggering inactive genes within heterochromatin.

### Neutrophils stimulated by IL-10, LPS, or TNF-regulated gene-specific profiles of PTMs *via* H3K4me3

In our previous studies, using H3K4me3 ChiP-Seq analysis, we identified various transcriptional start sites (TSSs) associated with the plasticity of human neutrophils exposed to TNF, IL-10, or directly stimulated by LPS. In this study, we have used our previously obtained data from ChiP-Seq of H3K4me3 to analyse target genes and gene ontology (GO) related to PTMs [9].

First, we have focused on the H3K4me3 peak breadth distribution, giving us an overall view of positioned genes ready for transcription and requiring additional regulatory transcription DNA binding proteins within the TSS regions. The ChiP-Seq analysis of H3K4me3-enriched nucleosomes allowed us to distinguish two types of peaks within the TSS regions: sharp-narrow peaks (< 1 kb) positioned near the TSS of Exon1, not requiring additional DNA binding proteins and broad-peaks (> 4 kb) being under control by them [38]. In our study, we have noted significant changes in peak distribution within H3K4me3 defined in MACS2 analysis. The average breadth of H3K4me3 peaks after TNF and IL-10 exposure was significantly shortened compared to non-stimulated or LPS-stimulated neutrophils (Supplementary Fig. 5A). The detailed analysis of several peaks, divided into ‘sharp’ and ‘broad’, indicated that this phenomenon resulted from significantly increased sharp peaks (Supplementary Fig. 5B). This data suggests that TNF pre-activation and repolarisation of neutrophils by IL-10 introduce them as highly specialised cells that can prepare specific genes for transcription.

**Fig. 4:**
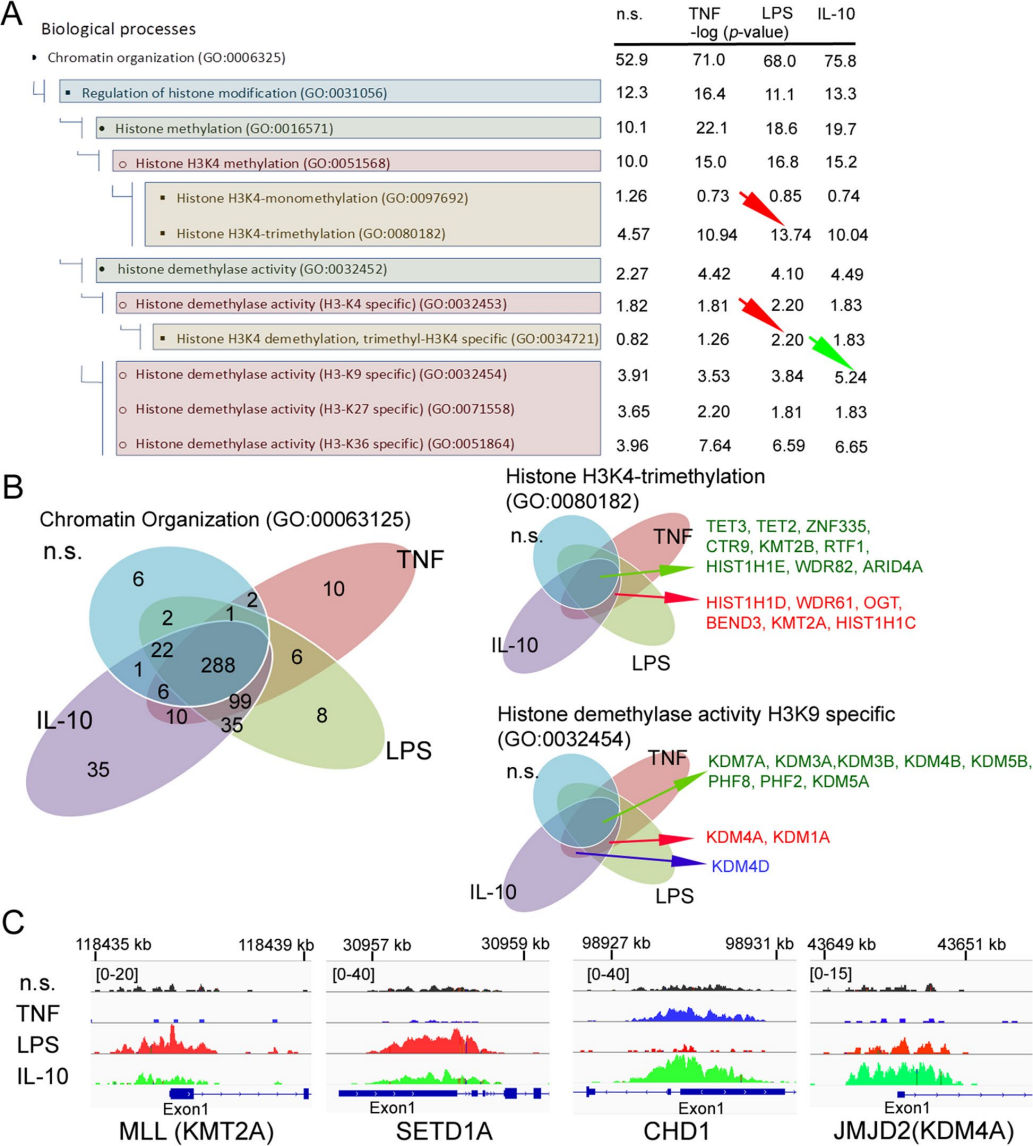
Neutrophils stimulated by IL-10, LPS, or TNF-regulated gene positioning specific profile of PTMs *via* H3K4me3 during different states of inflammation (pre-activation, activation or suppression). **A** Gene Ontology analysis of the Biological processes responsible for chromatin organisation mediated methylation and demethylation within histone H3 by positioning H3K4me3 methylation. Methylation of H3K4me3 is the process that dominates regardless of whether neutrophils are exposed to pro- or anti-inflammatory factors. Pro- vs. anti-inflammatory stimuli differences are determined *via* H3K4me3 ‘de positioning’ methylation of histone H3K9. The green arrow points to the demethylation of H3K9 as the process triggered upon exposure to IL-10. The red one points to H3K4me3 trimethylation as the parent process of neutrophil polarisation regardless of stimuli. Data are presented as mean values calculated from four independent experiments. **B** Binding site overlap in the GO term ‘Chromatin Organization’, ‘Histone H3K4-trimethylation’ and ‘Histone demethylase activity H3K9 specific’; n.s, non-stimulated. The analysis of target genes in the GO terms Chromatin organisation are presented in Supplementary Table 1. **C** The comparison of peak density within PTM enzymes revealed high signal within TSSs (Exon 1) of MLL1 and SETD1A in neutrophils stimulated by LPS and increased density within CHD1 as well as JMJD2A in neutrophils exposed to IL-10

To determine the effect of H3K4me3-marked histone on remodelling chromatin, we have performed a Gene Ontology analysis of processes engaged in PTMs. We have discovered that neutrophils, regardless of the type of stimuli used, are characterised by high positing ‘Chromatin organisation’ (GO:000631250) compared to non-stimulated neutrophils (Fig. 4A). The detailed analysis of target genes within this term revealed that there are 35 target genes, which are selectively positioned by IL-10 stimulation, 10 by TNF and 8 by LPS (Fig. 4B and Supplementary Table 1A). TNF pre-activated neutrophil, opposite to IL-10 or LPS stimulation, positioned genes within term related to histone acetylation: ‘Histone acetyltransferase complex’ (GO:0000123), ‘Histone acetyltransferase activity’ (GO:0004402) and ‘DNA-binding transcription factors’ (GO:0140297) (Supplementary Fig. 6). Detailed analysis of particular genes within terms ‘Histone acetyltransferase complex (GO:0000123)’ and ‘Histone methyltransferase complex’ (GO:0035097) revealed that more genes are positioned by H3K4me3 regardless of the stimuli used (Supplementary Table 1B). In turn, LPS stimulation was characterised by positioning target genes within two processes: ‘Histone H3K4-trimethyltransferase activity’ (GO:0080182) and ‘Histone H3K4 demethylase trimethyl H3K4 specific’ (GO:0034721). The comprehensive analysis within the term ‘Histone H3K4-trimethylation’ highlighted that all target genes are located in the common set (9 from 15 all in term) and 6, which characterised activated neutrophils regardless of the kind of stimuli (Fig. 4B right graphs). Contrary to LPS or TNF, IL-10 stimulated positioning target genes within terms ‘Histone demethylase activity H4K9 specific’ (GO:0032454) (Fig. 4A and Supplementary Fig. 6). The detailed analysis within this term highlighted that all target genes are located in the common set (8 from 11 all in term), 2 are characterised by positioning activated neutrophils regardless of stimulation (Fig. 4B) and one: KDM4D specific form for IL-10 or TNF exposition.

Next, we have focused on the ChiP-Seq H3K4me3 peak density readings within the TSS region of genes MLL1, SETD1A, CHD1 and JMJD2A. LPS-stimulated neutrophils, contrary to ‘n.s.’, IL-10, TNF exposition was characterised by high peak density within Exon 1 coded MLL1 and SETD1A. Neutrophils exposed to IL-10 were characterised by high peak density within CHD1 and JMJD2A (Fig. 4C). ChiP-Seq, together with ICC and mRNA analysis of these enzymes, revealed an overriding mechanism related to neutrophil polarisation during different stages of inflammation.

IL-10 stimulation triggered gene positioning *via* H3K4me3 and coded CHD1 and JMJD2A- enzymes remodelling chromatin to initiate transcription of gene hidden within heterochromatin and/or marked by H3K9me3. Contrary to IL-10, LPS stimulation does not require such chromatin rearrangement; genes positioned by H3K4me3 coded methyltransferase enzymes amplify anti-microbial properties by direct gene positioning within H3K4me3 (probably in a positive feedback manner). In turn, TNF-preactivation prepares neutrophils for a more effective response against pathogens by engaging target genes associated with binding transcription factors to DNA and target genes within acetylation of H3K27ac responsible for positioning genes susceptible to enhancers.

### Validation of *In Vitro* data to clinical status

Finally, we have verified the obtained data by their extrapolation to adequate clinical status, using neutrophils derived from patients with sepsis (systemic septic inflammation with LPS-stimulated neutrophils), NMOSD (aseptic inflammation with pre-activated neutrophils), and periodontitis (local self-limiting septic inflammation with IL-10-positive neutrophils).

**Fig. 5:**
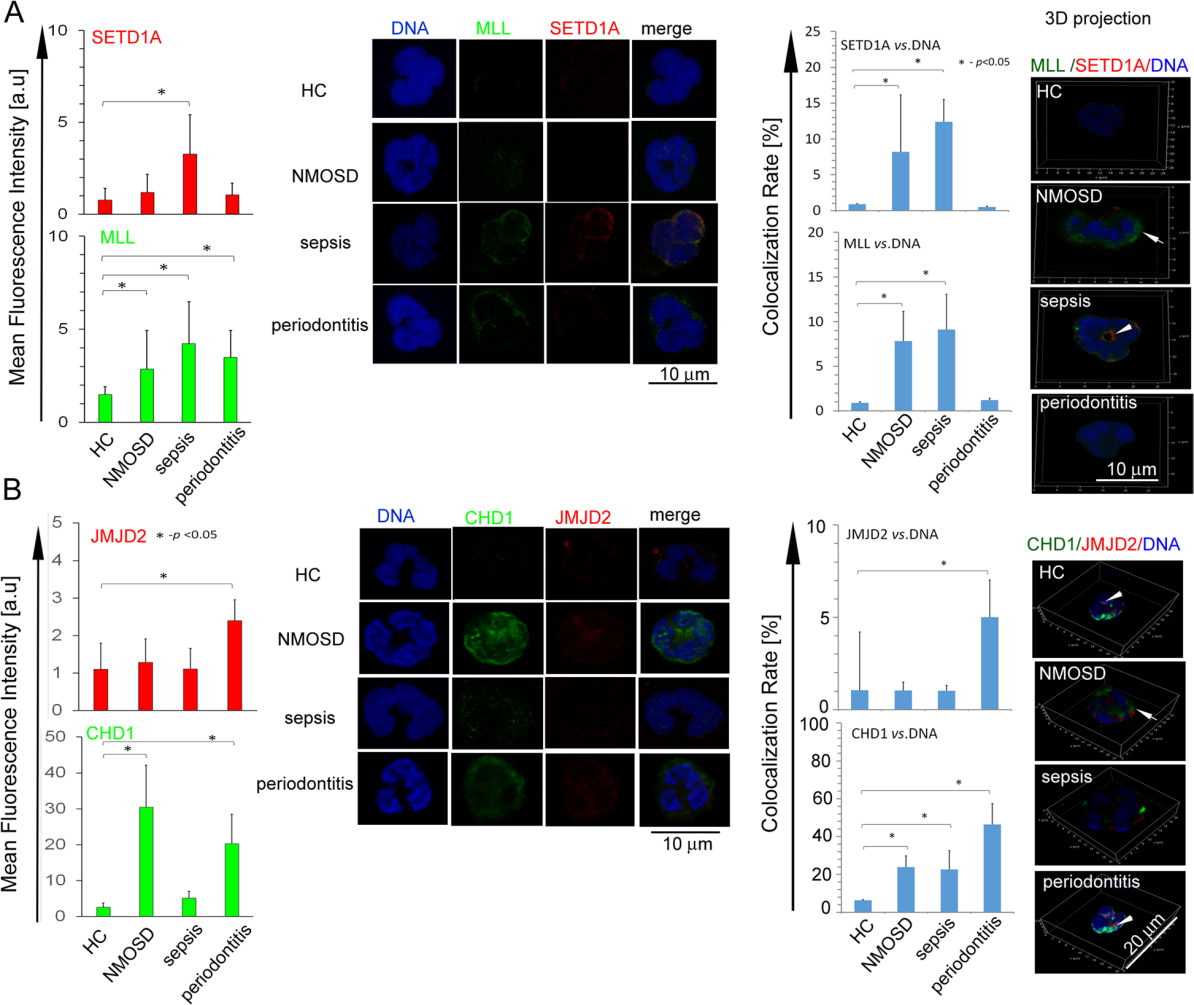
**A** Circulating sepsis neutrophils, similar to the in vitro LPS model, induced inflammation characterised by high expression of SETD1A and MLL1 and their colocalisation to the nucleus. **B** In turn, periodontitis neutrophils, similar to the in vitro IL-10 model, stimulated HC neutrophils with high expression of JMJD2A and CHD1 (A and B upper panels). The example of ICC analysis and confocal 3D projection confirmed colocalisation of SETD1A and MLL1 within the nucleus in sepsis patients, while colocalisation of JMJD2A and CHD1 within the nucleus mainly in periodontitis patients (white arrows) (A and B right panels). Data are presented as mean ± SD calculated based on 3 cases of patients with NMOSD, 6 with sepsis and 12 with periodontitis

In the first comparison, ICC analysis of enzyme expression has shown a correspondence between the in vitro model and a particular disease. SETD1A and MLL1 revealed a high expression and colocalisation within DNA in sepsis patients, corresponding with the in vitro model of LPS-stimulated neutrophils (Fig. 5A). In turn, neutrophils isolated from periodontitis were characterised by high expression of CHD1 and JMJD2A, which corresponds with the in vitro model of neutrophils exposed to IL-10 (Fig. 5B). Surprisingly, we have also observed high expression of MLL1 in NMOSD and periodontitis patients, but without explicit colocalisation within DNA, which may be related to MLL1 and SETD1A synthesis, but with limited capacity to colocalisation into cell nucleus (Fig. 5A, right panel).

**Fig. 6:**
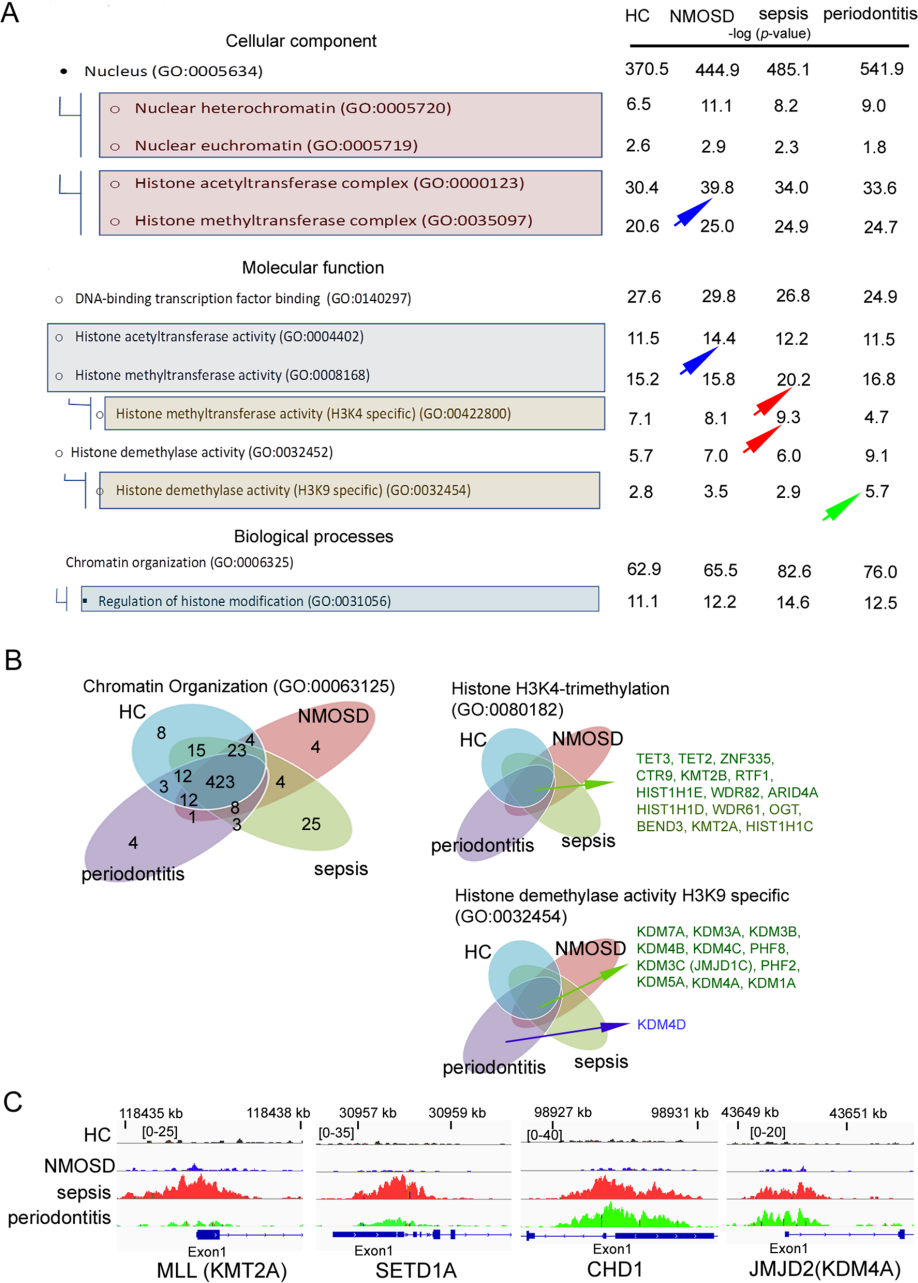
**A** Gene Ontology analysis of neutrophils isolated from NMOSD, sepsis and periodontitis patients confirmed that H3K4me3 positions target genes within the main processes associated with chromatin organisation. NMOSD neutrophils are characterised by positioning target genes related to histone acetyltransferase activity that corresponds with TNF-preactivated neutrophils (blue arrows), sepsis neutrophils positioned target genes associated with histone methyltransferase, especially H3K4 specific what correspond with LPS-stimulated neutrophils (red arrows). In turn, periodontitis neutrophils positioned target genes within the term ‘Histone demethylase activity’, particularly H3K9 (green arrows), which correlates with IL-10-stimulated neutrophils. **B** Binding site overlap in the GO terms ‘Chromatin organisation’, ‘Histone H3K4 trimethylation’ and ‘Histone demethylase activity H3K9 specific’. **C** The comparison of peak density within PTM enzymes revealed high signal within TSSs (Exon 1) of MLL1 and SETD1A in sepsis circulating neutrophils, high density within CHD1 as well as JMJD2A in periodontitis and sepsis which reflects in vitro model of IL-10-stimulated, but not in LPS-stimulated neutrophils

Next, we have focused on GO processes positioned by H3K4me3 and associated with chromatin organisation that emerged in vitro experiments. We have noted that high positioning target genes characterised neutrophils isolated from patients with NMOSD within terms ‘Histone acetyltransferase complex’ (GO:0000123) and ‘Acetyltransferase activity’ (GO: 0004402), which corresponds to TNF-preactivated neutrophils. In turn, neutrophils isolated from sepsis patients highly positioned genes associated with ‘Histone methyltransferase activity’ (GO:0008168), in particular, ‘Histone methyltransferase activity H3K4me3 specific’ (GO:00422800), which corresponds with in vitro model of LPS-stimulated neutrophils. Similarly, neutrophils isolated from patients with periodontal diseases highly positioned genes associated with ‘Histone demethylase activity’ (GO:0032454), particularly ‘H3K9 specific’ (GO:0031056). Regardless of the type of disease, all clinical groups were characterised by high positioning of target genes determined by ‘Chromatin organisation’ (GO:0006325) (Fig. 6A). Although we noted a high correlation in this point of GO analysis, the particular target gene analysis within this term shows significant differences compared to the in vitro model. The vena plot analysis within terms: ‘Chromatin organisation’, ‘Histone H3K4me3 trimethylation’ (GO:0080182) and ‘Histone demethylase activity H3K9 specific’ (GO:0032454)’ revealed that more target genes are located in the common compartments representing target gene positioning whether the neutrophils are isolated from HC, NMOSD, sepsis or periodontitis patients (Fig. 6B). Moreover, the compartment analysis within the ‘Chromatin organisation’ term also pointed to the target genes that are specific for NMOSD (4 genes), sepsis (25 genes) or periodontitis (4 genes) (Fig. 6B). Among these genes, we noted those that additionally correlate with the in vitro model: KDM4D (Lysine Demethylase 4D - JMJD2D) for periodontitis patients, AURKA (aurora kinase A) for NMOSD and HLTF (Helicase Like Transcription Factor) for sepsis patients (Supplementary Table 2 green font). In the next step of the bioinformatic analysis, we have focused on comparing peak density within TSSs (Exon 1) of PTM enzymes: MLL1, SETD1A, JMJD2A and CHD1. In this comparison, we have noted only partial correspondences with the in vitro inflammation model. Especially, the high signal of MLL1, SETD1A, but also CHD1 and JMJD2A is observed in the course of sepsis which did not correlate with LPS stimulation resulting in MLL1 and STED1A high signal only. Instead, we did not observe CHD1 in the course of NMOSD which did not correspond with TNF-preactivation in the in vitro model. This difference is probably due to the limitation of the inflammation model. In the course of sepsis, neutrophils are exposed to other factors, including pro-inflammatory cytokines, heat shock proteins, and complement components (C5a, C3a). These factors probably affect neutrophils simultaneously. In turn, in the course of NMOSD, neutrophils are preactivated mainly by C5a, but not TNF, which probably engages different pathways for their preactivation [21].

High density within CHD1 as well as JMJD2A is observed in periodontitis which accurately corresponds with the in vitro model- neutrophils exposed to IL-10 (Fig. 6C). To summarise, this data may suggest that changes within the target genes of these enzymes are quantitative; thus, gene expression depends on the number of positioned genes within DNA but is not associated with positioning the new one.

Finally, we have compared peak breadth distribution within genes positioned by H3K4me3, and we noted a decrease in the average breadth as well as more ‘sharp–narrow’ peaks in patients with NMOSD and periodontitis, which corresponds with TNF-preactivated and IL-10-stimulated neutrophils (Supplementary Fig. 5C, D).

In the last step of validating the in vitro model to adequate clinic status, we examined the level of two PTMs we had expected to be remarkably characterised by pre-activated neutrophils and these exposed to IL-10. For pre-activated neutrophils, we have examined the level of H3K27ac responsible for positioning enhancers. For IL-10 stimulation, we estimated the level of H3S10p, which, in turn, was related to triggering genes within heterochromatin. Due to the limited number of cells obtained from patients, we have used ICC despite the dot blot method. We have noted high expression of H3K27ac in neutrophils isolated from NMOSD patients, which characterised pre-activated neutrophils. In turn, neutrophils isolated from periodontitis were characterised by a high level of H3S10p, which corresponds with neutrophils exposed to IL-10 (Supplementary Fig. 7). In our previous investigation using the same technique, clinical samples and in vitro model, we had shown that neutrophils isolated from sepsis patients, opposite to HC, were characterised by a high level of H3K4me3, which corresponds with the in vitro model of LPS-stimulation [9].

## Discussion

One of the crucial questions raised in our research concerns the overriding mechanism that reflects the changeable nature of neutrophils from cells ready to neutralise pathogens to those reducing inflammation and initiating tissue regeneration after IL-10 stimulation. As we have shown in our previous studies, the neutrophil polarisation to immunosuppressive, restricted inflammatory cells is associated with initiating gene transcription dependent on alternative NF-kB pathway activation with simultaneous inhibition of classical NF-kB pathway activation. In the current research, we have shown that this process results from the reduction in the properties of neutrophils to E. coli phagocytosis, ROI production and CD11b/CD18 expression after TNF preactivation or LPS stimulation. In our opinion, it is physiologically justified, as maintenance of neutrophils’ anti-infection potential is high energy and unnecessary for cytokine/chemokine or growth factor synthesis during tissue regeneration [9]. We speculated that such a significant change, which completely alters their function, can go through the activation of primarily inactive genes. It should result from significant changes at the level of histone rearrangement, engaging chromatin remodelling proteins and gene silencing within heterochromatin. From the four protein subfamilies related to chromatin remodelling (ISWI, CHD, SWI/SNF and INO80), we designated chromodomain helicase DNA-binding protein 1 (CHD1) as the enzyme considered an effector of active histone modification by specific recognition of H3K4me3 residues produced by Set1 [39]. Therefore, CHD1 is regarded as a chromatin remodeler, strongly associated with transcription and nucleosome turnover downstream of the TSS [35]. Johnsen and colleagues show that CHD1 is required to induce osteoblast-specific gene expression, extracellular-matrix mineralisation and ectopic bone formation in vivo, which directed this molecule on the high potential in cell polarity [40]. In our study, we noted that neutrophils exposed to IL-10 were characterised by high CHD1 protein and mRNA expression and high NGS density reading within the TSS region of *CHD1* genes marked by H3K4me3. Another molecule that we suspected to be related to IL-10-induced neutrophil polarity is histone demethylase JMJD2A, also known as KDM4A, which promotes gene transcription by antagonising H3K9 specific Lys9 residues histone H3 methylation marked heterochromatin [41, 42]. Similarly to CHD1 analysis, we have noted that neutrophils exposed to IL-10 were characterised by high JMJD2A protein and mRNA expression as well as high NGS density reading within the TSS region of *JMJD2A* genes marked by H3K4me3. Opposite to CHD1, JMJD2A seems to be not involved in neutrophil TNF-preactivation.

LPS added to neutrophils previously exposed to IL-10 prevents inducing CHD1 and JMJD2A by IL-10. As both enzymes are- actively transcribed and require TSS positioning by H3K4me3, alteration within poisoning H3K4me3 by stimulating neutrophils with LPS blocks active transcription of CHD1 and JMJD2A by IL-10 stimulation. This hypothesis is also moderately supported by studies of circulating neutrophils isolated from sepsis, NMOSD and periodontitis patients. Neutrophils isolated from periodontitis that respond correctly to IL-10 were characterised by high CHD1 and JMJD2A expression (ICC and ChiP-Seq H3K4me3 results), opposite to sepsis-derived neutrophils, with no changes within these enzymes. We have also noted high CHD1 expression in NMOSD neutrophils, which corresponds with the result from the in vitro model of TNF pre-activation. Investigating further mechanism that prevents the action of IL-10 in an ongoing inflammatory reaction, we have drawn attention to PTMs induced by direct exposure to LPS. LPS-stimulated neutrophils, on the one hand, were characterised by lower levels of H3K9me3, H3K79me2 and H3K27me3- the profile of histone modification responsible for gene repression, on the other hand, by high levels of H3K4me3- PTMs, which position active transcribed genes within the end of Exon1. In our opinion, this is not a natural process of eliminating pathogens because first, these cells must be pre-activated by a low concentration of pro-inflammatory factors. The pre-activation process probably prevents neutrophils from being activated in the blood, gives additional time for chemotaxis, diapedesis, and enhances processes involved in antimicrobial activity. This theory is confirmed by our analysis of functional tests (ROI production and phagocytosis), where neutrophils entering blood vessels are already prepared for a response in contact with the pathogen; however, this response is less intense than before TNF-preactivation. Pathological, direct LPS activation can be observed in the periphery of sepsis patients. The analysis of neutrophils isolated from blood patients with sepsis revealed the correlation with the in vitro model within the expression of MLL1 and SETD1A, as well as analysis of GO processes positioned by H3K4me3 related to chromatin remodelling. In addition, we have also presented an in vitro model that direct LPS stimulation ‘blocks’ IL-10-induced suppression of ROI production, phagocytosis, CD11b/CD18 and CD62L adhesion molecules expression. A series of in vitro experiments and neutrophils isolated from blood sepsis patients prompted us to hypothesise that neutrophils directly exposed to LPS trigger the fast pathway of gene transcription through the involvement of H3K4me3-specific methyltransferases. This process is irreversible, avoiding chromatin rearrangement by IL-10. Probably, this phenomenon has severe clinical implications in the course of the second phase of sepsis, during compensatory anti-inflammatory response syndrome (CARS), as neutrophils isolated from the blood of these patients were characterised *inter alia*: abnormal high number immature neutrophils, delayed apoptosis and uncontrolled synthesis of pro-inflammatory factors despite of high concentration of anti-inflammatory IL-10 in serum [43, 44].

On the other hand, the proper reaction associated with eliminating pathogens preceded by pre-activation can also lead to pathological conditions. In the course of autoimmune diseases such as NMOSD, diabetes mellitus type 1 (DM1), and rheumatoid arthritis (RA), circulating neutrophils are permanently pre-activated with enhanced properties to phagocytosis, ROI production, high cell surface adhesion of molecule expression in ex vivo stimulation as well as prolongated life spine. Similar to the case of sepsis, the high concentration of IL-10 observed in the neutrophil environment, does not prevent their preactivation and its negative effects. A high concentration of anti-inflammatory IL-10 is observed in serum rheumatoid arthritis RA; high in serum and cerebrospinal fluid in NMOSD, high in culture supernatants LPS-stimulated neutrophils isolated from DM1 patients [45–47]. This study revealed that H3K4me1 and H3K27ac, which characterised patterns of PTMs for enhancers, mark TNF-preactivated neutrophils. Furthermore, we have shown that the process of H3K27 acetylation (H3K27ac), but not H3K4 monomethylation (H3K4me1), is triggered by positioning genes within H3K4me3. We concluded that H3K27ac enables neutrophils to have a much stronger and more targeted cellular response to proinflammatory cytokines arriving at the site of inflammation. This thesis, based on an in vitro model pre-activation, is reflected in neutrophils isolated from blood NMOSD patients which are characterized by high levels of H3K27ac.

IL-10-stimulated neutrophils are characterised by PTM-marked active promotors, transcribed regions and enhancers. Although this modification sometimes coincides with pre-activation or LPS activation, the level of IL-10-induced PTMs is the most diverse. Opposite to TNF or LPS, it is additionally characterised by high phosphorylation of histone H3 at serine 10 (H3S10p). H3S10 phosphorylation is involved in chromatin compaction during mitosis, meiosis, and chromatin relaxation after transcription activation [48]. Although H3S10P is referred to as a marker of mitosis, it can also function as an ‘open-chromatin factor’ in interphase for a subset of genes, allowing many different elements to access the chromatin, keeping them in a more open state to enable transcription [49, 50]. The H3S10p cooperated with histone H3 at lysine 9 (H3K9me3) *via* heterochromatin protein beta (HP1b), a family of heterochromatic adaptor molecules implicated in both gene silencing and supra-nucleosomal chromatin structure during mitosis. This inhibition phenomenon of the ability of H3K9me3 to bind HP1b by H3S10p is responsible for heterochromatin reorganisation [51]. As in our experiment, we have observed high levels of H3K9me3 and H3S10p in IL-10-stimulated neutrophils, and we speculated that this modification could also be responsible for the activation of genes ordinarily unavailable during cell activation by proinflammatory factors. This phenomenon is probably essential for protecting tissue regeneration by restricting inappropriate inflammation-promoting genes.

The neutrophil TNF-preactivation or IL-10 stimulation results in a specific-ultimate gene reorganisation for transcription also reflected in peak lengths within Exon 1 of H3K4me3 histone. H3K4me3-marked nucleosomes are detected as sharp-narrow peaks flanking the TSSs at the end of the first exon, strictly correlating with transcriptional activity. In our study, we have revealed that this kind of peak significantly increases with TNF pre-activation or after IL-10 exposure, which, in our opinion, proves the extremely polarised state of neutrophils. In turn, unstimulated neutrophils characterised by H3K4me3-marked nucleosomes were detected as broad peaks associated with many histone modifications. Genes in broad H3K4me3 peaks are involved in cell plasticity and their polarisation to specialised cells. This process is responsible for the adaptation of the cell to changing environmental conditions and seems critical in the polarisation of immunocompetent cells during inflammation. The broad peaks within H3K4me3 are the purpose of the action of enhancers, super-enhancers and transcription factors [38]. Therefore, the large number of broad H3K4me3 peaks proves that resting neutrophils can make rapid polarisation in one of the two possible opposite manner: maximising response to pathogens initiated by TNF-preactivation or starting neutrophil pro-tissues regeneration triggering by IL-10. On the other hand, we did not observe any changes within H3K4me3 peak distribution in LPS-stimulated neutrophils, which can indicate that neutrophils passing into the bloodstream do not require such changes within H3K4me3. This hypothesis is reflected in the clinic, the average breadth and number peaks of H3K4me3 decrease in NMOSD, and there is no difference in sepsis patients within breadth peaks of H3K4me3.

The significant role of nuclear chromatin modifying enzymes during various periods of inflammation is evidenced by paired comparison to monocytes as the immune cells with shared ontology [52]. The comparative analysis of eQTL (expression quantitative trait loci) maps of neutrophils and monocytes from 4 to 8 individuals by the BLUEPRINT consortium, revealed that they have substantial overlap in regions of the genome that are methylated and similar profile histone modifications [52, 53]. The differences in gene expression within both populations probably result from differences within enhancers and PTM enzymes. This hypothesis is confirmed by research with the ontology approach. J. Knight groups analysed enhancer maps generated by CAGE-Seq in FANTOM [54, 55] and revealed that neutrophils opposite to monocytes are much more enriched in the enhancer region [52], therefore they are more sensitive to environmental change and adapting to it more variously. These data also elucidate the phenomenon that both cells in similar environments, at the same time, exert different biological effects within periods of immunity. This phenomenon is additionally confirmed in the clinic. The same group of scientists revealed that the allele at rs2240335 within PADI4 affects rheumatoid arthritis susceptibility by increased expression of PADI4 in neutrophils but reduced in monocytes. This opposite mRNA expression between both leukocytes is the result of the marked region within PADI4 by H3K27ac and H3K4me1 in neutrophils but not in monocytes [52]. These studies confirm our hypothesis that in autoimmune diseases permanently preactivated neutrophils which are characterized by a high level of H3K27ac and H3K4me1. Although we used neutrophils isolated from patients suffering from NMOSD as an example of autoimmune disease, we also noted high levels of H3K27ac. As we presented in our other studies, NMOSD C5a-preactivated neutrophils reverse astrocyte glutamate pump into extracellular space, as a consequence of pathological glutamate removal from extracellular space [21].

## Conclusions

Our investigation, directed at two main phenomena, brings us closer to understanding the physiological nature of neutrophils in tissue regeneration and their pathological role in sepsis and autoimmune diseases. First, the profile of PTMs determined the actual status of neutrophils during infection. Newly emerging neutrophils in the periphery are characterised by constitutively high expression of H3K4me3, which possess TSS regions associated with antimicrobial properties and genes coding histone PTM enzymes responsible for adapting neutrophils to the current inflammation state. Second, IL-10-induced JMJD2A and CHD1 can encourage genes desired in the regeneration of tissues after infection and prevent neutrophils from LPS-activation or TNF-preactivation. This phenomenon elucidates that the signal from the inflammation site can completely change the character from pre-prepared to neutralise pathogens with high antimicrobial potential into neutrophils with desired properties in tissue regeneration. LPS stimulation triggers a positive feedback mechanism that enhances the synthesis of enzymes that maintain histone-H3-lysine-4 methylation on transcriptionally active promoters (SETD1A and MLL1) by positioning H3K4me3 itself. Because transcription of JMJD2A and CHD1 also depends on TSS positioning by H3K4me3, neutrophils before LPS stimulation become insensitive to IL-10. The second conclusion is that these changes are irreversible. Neutrophils pre-activated by TNF or directly stimulated by LPS become insensitive to the anti-inflammatory effects of IL-10, and vice versa; IL-10 protects neutrophils against proinflammatory stimuli. There are practical implications in a new treatment approach to patients with sepsis, that should take into account medical interventions already at the stage of myelopoiesis before neutrophil exposure to LPS.

### Supplementary Information

Supplementary Material 1: Supplementary Figure 1. IL-10 protects neutrophils against adhesion, transendothelial migration and preactivation provided they had not been previously exposed to TNF or LPS.  (A, B) The direct exposition of TNF, LPS, IL-10 on β2 integrins (CD11b/CD18) expression and the effect of IL-10 on the neutrophil TNF pre- and LPS-activation process. The right part of the upper graphs (demarcated by a dashed line) demonstrates the protective effect of IL-10 against TNF-preactivation or LPS-activation as well as the disruption of this process due to previous short-term exposure to TNF or LPS. (C) The analysis of direct exposition of TNF, and LPS on the shadings of L-selectin (CD62L) and the protective effect of IL-10 on this phenomenon. The right part of the upper graph (demarcated by a dashed line) demonstrates the protective effect of IL-10 on TNF-pre-activated and LPS-activated neutrophils and the disruption of this process due to previous short-term exposure to TNF or LPS.  The low panels of Fig. A, B and C present the most representative examples of three independent experiments.

Supplementary Material 2: Supplementary Figure 2. Patterns of selected posttranslational histone modifications characterised neutrophils after TNF-preactivation and subsequent activation by LPS as an *in vitro* model of physiological neutrophil activation at the site of inflammation (left panel of each graph) and the effect of IL-10 on this process (right panel of each graph). To demonstrate the protective IL-10 effect on TNF-preactivation and LPS stimulation, neutrophils were exposed to different orders of IL-10. Average level ± SD of posttranslational histone modifications performed on four independent experiments. Statistical significance was compared between ‘n.s.’ *vs.* TNF+LPS and IL-10 in different TNF or LPS exposure orders. Active promotors are signed in green, transcribed regions in purple, repression genes in red, and enhancer regions in blue colour (upper panel). The low panel presents the example of dot blot analysis.

Supplementary Material 3: Supplementary Figure 3. LPS-stimulated neutrophils, opposite to IL-10 or TNF, were characterised by high colocalisation of MLL1 and SETD1A within the nucleus (A left panel). IL-10 inhibits the appearance of MLL1 and SETD1A within DNA during LPS stimulation, but only if IL-10 acts prior to LPS stimulation (A right panel). (B) Example of MLL1* vs*. DNA and SETD1A *vs*. DNA colocalisation analysis. (C) 3D projection confirms high expression of MLL1 and SED1A in the nucleus stimulated by LPS, contrary to TNF or IL-10 stimulation (white arrows indicate areas of colocalisation).

Supplementary Material 4: Supplementary Figure 4. IL-10-stimulated neutrophils, opposite to LPS or TNF, were characterised by high colocalisation of CHD1 and JMJD2A within the nucleus (A left panel on each graph). Previous LPS stimulation disturbs CHD1 colocalisation induced by IL-10 (inhibits the appearance of CHD1 within DNA) (A left panel). Prior TNF-preactivation or LPS stimulation disrupts IL-10-induced JMJD2A colocalisation within DNA (A left panel). (B) Example of JMJD2A* vs* DNA and CHD1A *vs* DNA colocalisation analysis during TNF-preactivation, LPS- or IL-10 stimulation. (C) 3D projection confirms the disturbing colocalisation of CHD1A prior to LPS stimulation and JMJD2A colocalisation before TNF or LPS stimulation (white arrows indicate areas of colocalisation).

Supplementary Material 5: Supplementary Figure 5. The statistical analysis of ChiP-Seq H3K4me3 peak breadth distribution in non- and stimulated neutrophils by TNF-a, LPS or Il-10 as *in vitro* model of different states during inflammation and adequate clinical status. (A) The statistical analysis of ChiP-Seq H3K4me3 peak breadth distribution pointed to a reduction of average breadth and number of peaks during TNF-preactivation. (B left panel) The study of the breadth peaks revealed that TNF-preactivated and IL-10-stimulated neutrophils are characterised by increased ‘sharp narrow’ peaks (<1kb) compared to ‘n.s.’ or LPS stimulation. (B right panel) TNF-preactivation of neutrophils also reduces‘broad’ peaks (>4kb). (C) The ChiP-Seq H3K4me3 peak distribution statistical analysis revealed a decrease in average breadth and peak number in NMOSD patients, corresponding with TNF-preactivated neutrophils. (D) NMOSD and periodontitis neutrophils were characterised by an increased number of ‘sharp narrow’ peaks within genes positioned by H3K4me3 compared to HC or sepsis patients, which corresponds with TNF-preactivated and IL-10-stimulated neutrophils with adequate *in vitro* model.

Supplementary Material 6: Supplementary Figure 6. Neutrophils stimulated by IL-10, LPS, or TNF regulated gene profile associated with nucleus plasticity *via* H3K4me3-marked histone. (A) Regardless of stimuli, Gene Ontology within H3K4me3 target genes revealed a strong association with ‘Nuclear heterochromatin’ (GO:0005720). This process is associated with the forming of acetyltransferase and methyltransferase complexes. (B) Gene Ontology Molecular function highlighted significant variability within different stimuli. TNF-preactivation was characterised by positioning genes related to methyltransferase activity H3K4 and H3K27 specific, DNA-binding transcription factor, and acetyltransferase activity (blue arrows). In turn, LPS or IL-10-activated neutrophils by positioning genes associated with methyltransferase activity also H3K4 and H3K27 specific, but opposite to LPS-, IL-10-stimulated neutrophils additionally positioned genes associated with histone demethylase activity H3K9 specific (green and red arrow).

Supplementary Material 7: Supplementary Figure 7. Chromatin H3K27ac marked characterised pre-activated neutrophils observed in the course of autoimmune disease such as NMOSD, while H3S10p posttranslational histone modification is one of four characteristics for polarisation of neutrophils into resolving inflammatory cells induced by IL-10, physiologically observed in periodontological patients. (A and B left panels) The Mean Fluorescence Intensity statistical comparison was calculated based on 3 cases of patients with NMOSD, 6 with sepsis and 12 with periodontitis.

Supplementary Material 8: Supplementary Table 1 (A, B). The list of target genes in the GO terms:‘Chromatin organisation’, ‘Histone acetyltransferase complex’, and ‘Histone methyltransferase complex’ is divided into logical subsets specific for non-, LPS, TNF, or IL-10-stimulated neutrophils.

Supplementary Material 9: Supplementary Table 2. The list of target genes in the GO term‘Chromatin organisation’ is divided into logical subsets specific for HC, sepsis, NMOSD and periodontitis-derived neutrophils. Green font marked target genes specific for the clinic status of neutrophils (periodontitis, NMOSD and sepsis patients) and correspond with the adequate *in vitro* model.

## Data Availability

All relevant data are reported in the manuscript. The raw datasets used and analysed during the current study are available from the corresponding author upon request. Results of ChiP-Seq studies are available in the repository on the NCBI PubMed website (GEO https://www.ncbi.nlm.nih.gov/geo/query/acc.cgi?acc=GSE186508).
