# Supplementary figures and images for: Histone H3 posttranslational modified enzymes defined neutrophil plasticity and their vulnerability to IL-10 in the course of the inflammation
Source: J Inflamm (Lond). 2024 May 14;21:16. doi: 10.1186/s12950-024-00389-8 (PMC11095086; doi:10.1186/s12950-024-00389-8)

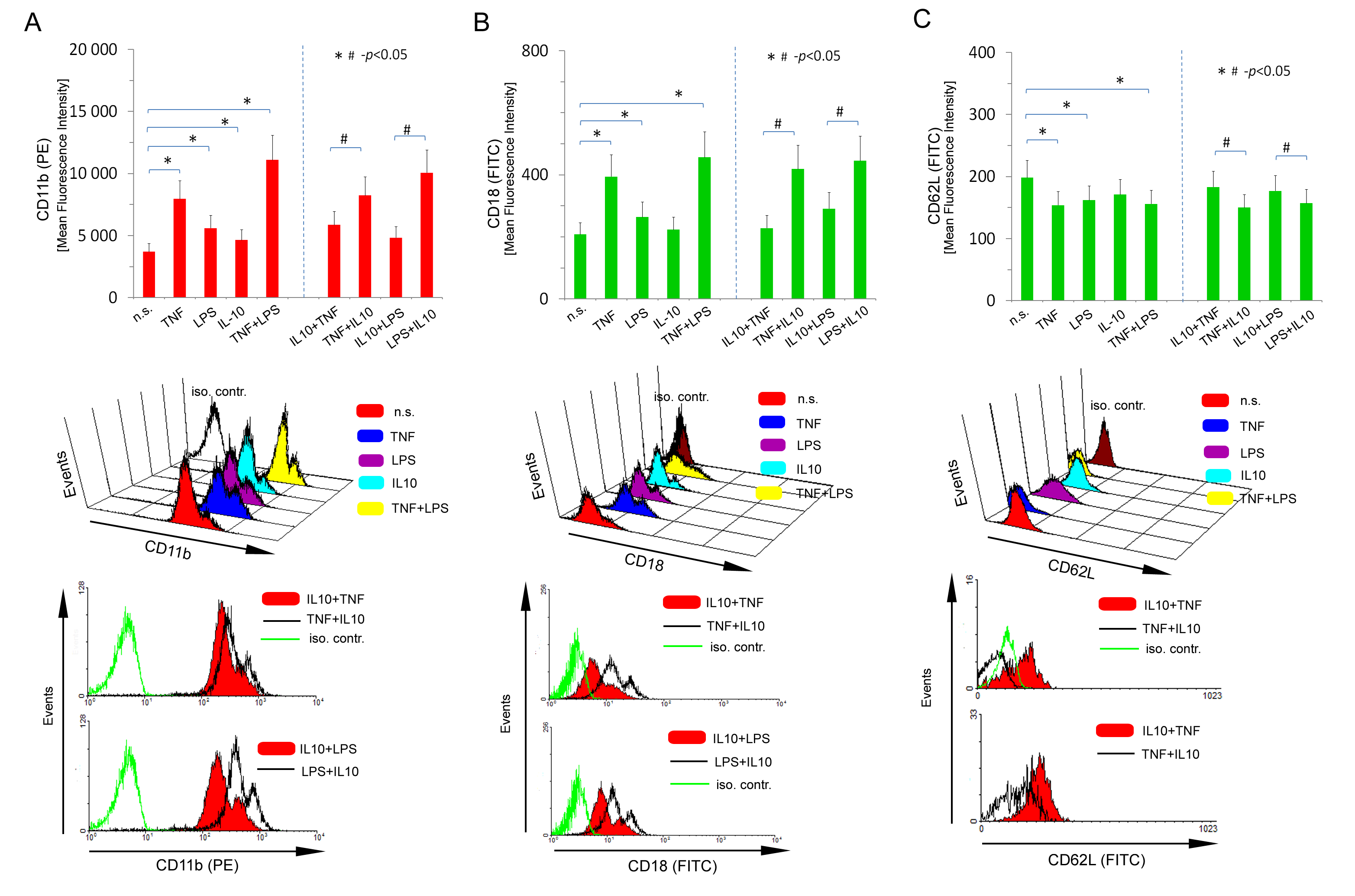

Supplement: Supplementary file 1 — Supplementary Material 1: Supplementary Figure 1. IL-10 protects neutrophils against adhesion, transendothelial migration and preactivation provided they had not been previously exposed to TNF or LPS. (A, B) The direct exposition of TNF, LPS, IL-10 on β2 integrins (CD11b/CD18) expression and the effect of IL-10 on the neutrophil TNF pre- and LPS-activation process. The right part of the upper graphs (demarcated by a dashed line) demonstrates the protective effect of IL-10 against TNF-preactivation or LPS-activation as well as the disruption of this process due to previous short-term exposure to TNF or LPS. (C) The analysis of direct exposition of TNF, and LPS on the shadings of L-selectin (CD62L) and the protective effect of IL-10 on this phenomenon. The right part of the upper graph (demarcated by a dashed line) demonstrates the protective effect of IL-10 on TNF-pre-activated and LPS-activated neutrophils and the disruption of this process due to previous short-term exposure to TNF or LPS. The low panels of Fig. A, B and C present the most representative examples of three independent experiments. [file 12950_2024_389_MOESM1_ESM.tif]

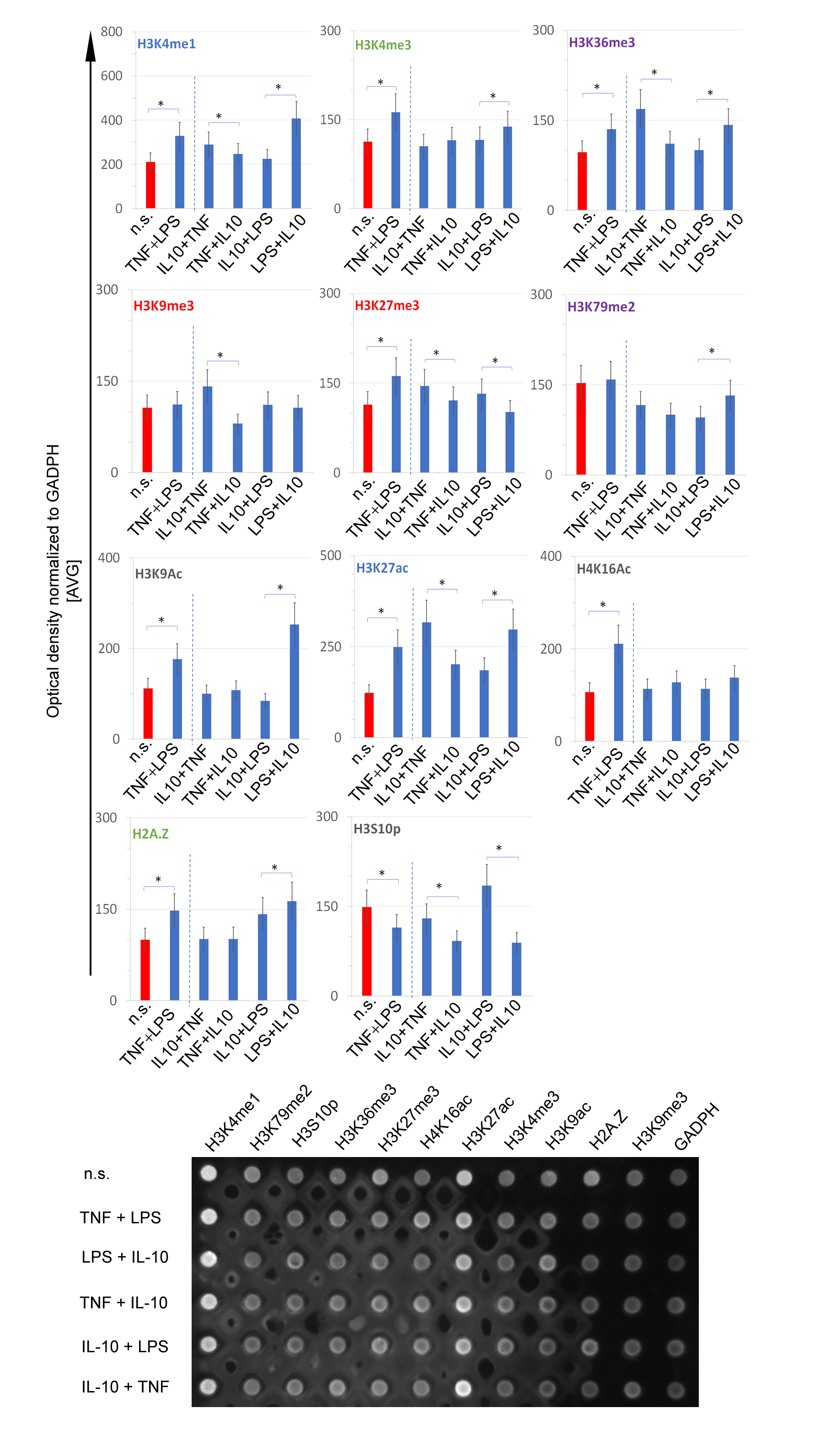

Supplement: Supplementary file 2 — Supplementary Material 2: Supplementary Figure 2. Patterns of selected posttranslational histone modifications characterised neutrophils after TNF-preactivation and subsequent activation by LPS as an in vitro model of physiological neutrophil activation at the site of inflammation (left panel of each graph) and the effect of IL-10 on this process (right panel of each graph). To demonstrate the protective IL-10 effect on TNF-preactivation and LPS stimulation, neutrophils were exposed to different orders of IL-10. Average level ± SD of posttranslational histone modifications performed on four independent experiments. Statistical significance was compared between ‘n.s.’ vs. TNF+LPS and IL-10 in different TNF or LPS exposure orders. Active promotors are signed in green, transcribed regions in purple, repression genes in red, and enhancer regions in blue colour (upper panel). The low panel presents the example of dot blot analysis. [file 12950_2024_389_MOESM2_ESM.tif]

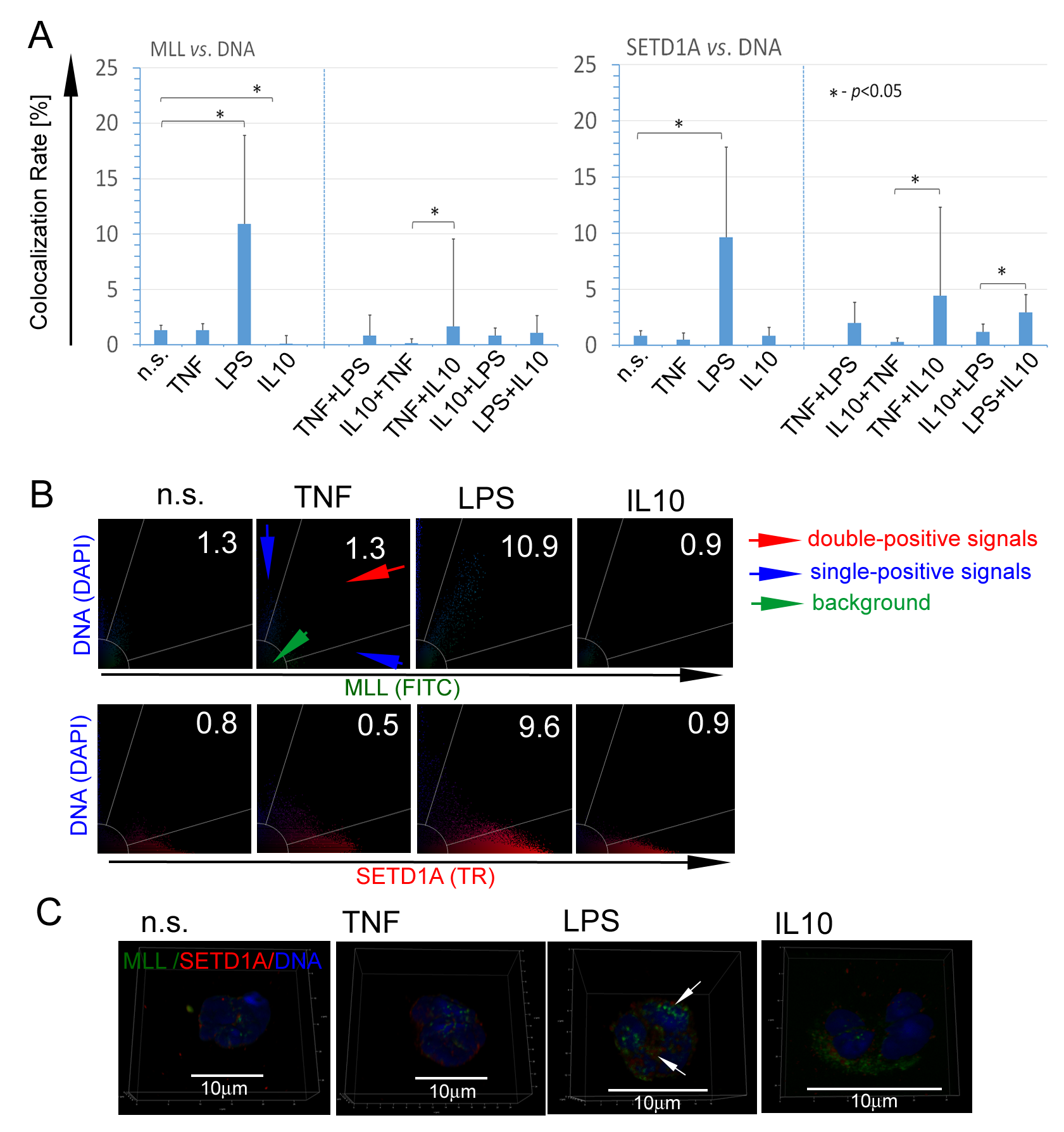

Supplement: Supplementary file 3 — Supplementary Material 3: Supplementary Figure 3. LPS-stimulated neutrophils, opposite to IL-10 or TNF, were characterised by high colocalisation of MLL1 and SETD1A within the nucleus (A left panel). IL-10 inhibits the appearance of MLL1 and SETD1A within DNA during LPS stimulation, but only if IL-10 acts prior to LPS stimulation (A right panel). (B) Example of MLL1 vs. DNA and SETD1A vs. DNA colocalisation analysis. (C) 3D projection confirms high expression of MLL1 and SED1A in the nucleus stimulated by LPS, contrary to TNF or IL-10 stimulation (white arrows indicate areas of colocalisation). [file 12950_2024_389_MOESM3_ESM.tif]

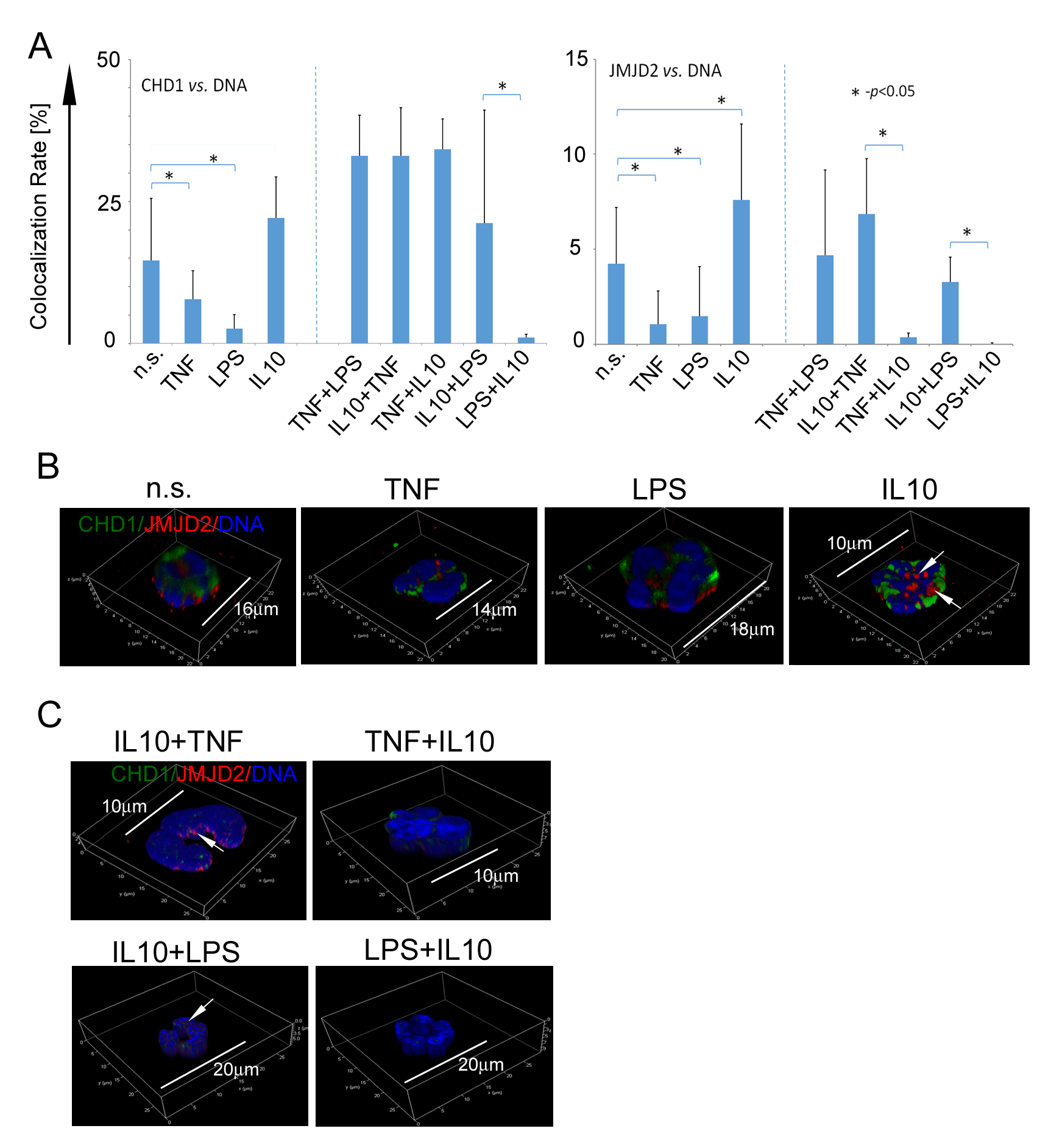

Supplement: Supplementary file 4 — Supplementary Material 4: Supplementary Figure 4. IL-10-stimulated neutrophils, opposite to LPS or TNF, were characterised by high colocalisation of CHD1 and JMJD2A within the nucleus (A left panel on each graph). Previous LPS stimulation disturbs CHD1 colocalisation induced by IL-10 (inhibits the appearance of CHD1 within DNA) (A left panel). Prior TNF-preactivation or LPS stimulation disrupts IL-10-induced JMJD2A colocalisation within DNA (A left panel). (B) Example of JMJD2A vs DNA and CHD1A vs DNA colocalisation analysis during TNF-preactivation, LPS- or IL-10 stimulation. (C) 3D projection confirms the disturbing colocalisation of CHD1A prior to LPS stimulation and JMJD2A colocalisation before TNF or LPS stimulation (white arrows indicate areas of colocalisation). [file 12950_2024_389_MOESM4_ESM.tif]

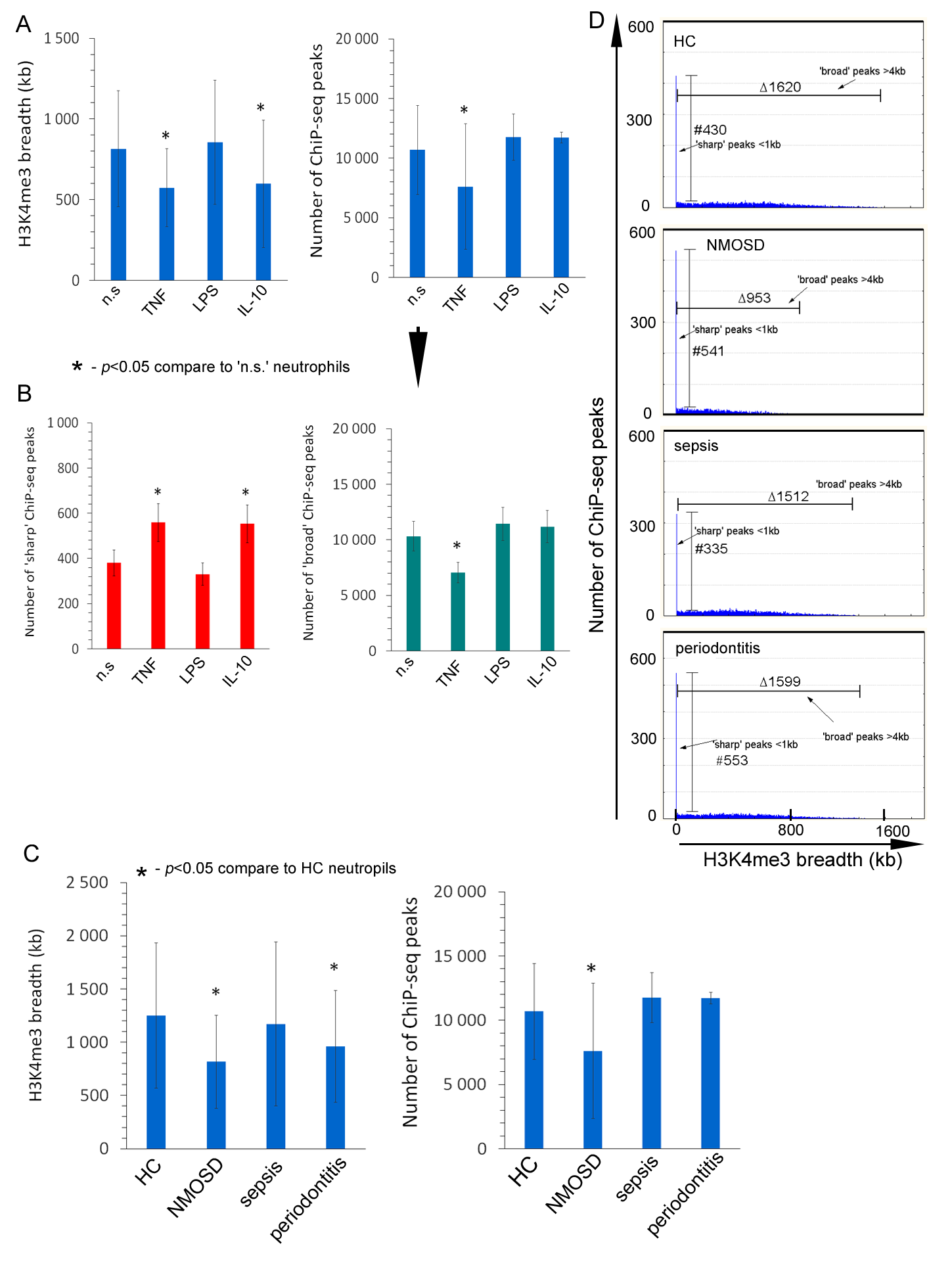

Supplement: Supplementary file 5 — Supplementary Material 5: Supplementary Figure 5. The statistical analysis of ChiP-Seq H3K4me3 peak breadth distribution in non- and stimulated neutrophils by TNF-a, LPS or Il-10 as in vitro model of different states during inflammation and adequate clinical status. (A) The statistical analysis of ChiP-Seq H3K4me3 peak breadth distribution pointed to a reduction of average breadth and number of peaks during TNF-preactivation. (B left panel) The study of the breadth peaks revealed that TNF-preactivated and IL-10-stimulated neutrophils are characterised by increased ‘sharp narrow’ peaks (<1kb) compared to ‘n.s.’ or LPS stimulation. (B right panel) TNF-preactivation of neutrophils also reduces‘broad’ peaks (>4kb). (C) The ChiP-Seq H3K4me3 peak distribution statistical analysis revealed a decrease in average breadth and peak number in NMOSD patients, corresponding with TNF-preactivated neutrophils. (D) NMOSD and periodontitis neutrophils were characterised by an increased number of ‘sharp narrow’ peaks within genes positioned by H3K4me3 compared to HC or sepsis patients, which corresponds with TNF-preactivated and IL-10-stimulated neutrophils with adequate in vitro model. [file 12950_2024_389_MOESM5_ESM.tif]

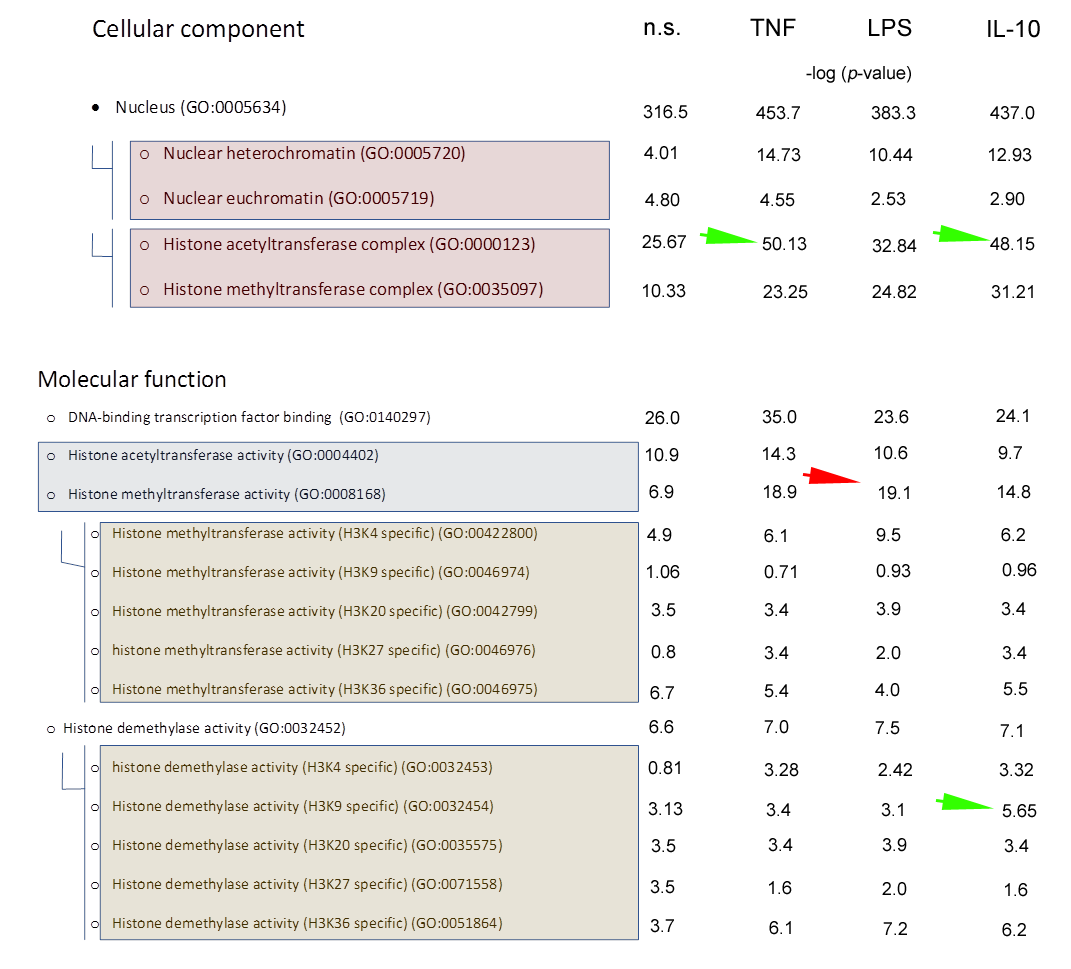

Supplement: Supplementary file 6 — Supplementary Material 6: Supplementary Figure 6. Neutrophils stimulated by IL-10, LPS, or TNF regulated gene profile associated with nucleus plasticity via H3K4me3-marked histone. (A) Regardless of stimuli, Gene Ontology within H3K4me3 target genes revealed a strong association with ‘Nuclear heterochromatin’ (GO:0005720). This process is associated with the forming of acetyltransferase and methyltransferase complexes. (B) Gene Ontology Molecular function highlighted significant variability within different stimuli. TNF-preactivation was characterised by positioning genes related to methyltransferase activity H3K4 and H3K27 specific, DNA-binding transcription factor, and acetyltransferase activity (blue arrows). In turn, LPS or IL-10-activated neutrophils by positioning genes associated with methyltransferase activity also H3K4 and H3K27 specific, but opposite to LPS-, IL-10-stimulated neutrophils additionally positioned genes associated with histone demethylase activity H3K9 specific (green and red arrow). [file 12950_2024_389_MOESM6_ESM.tif]

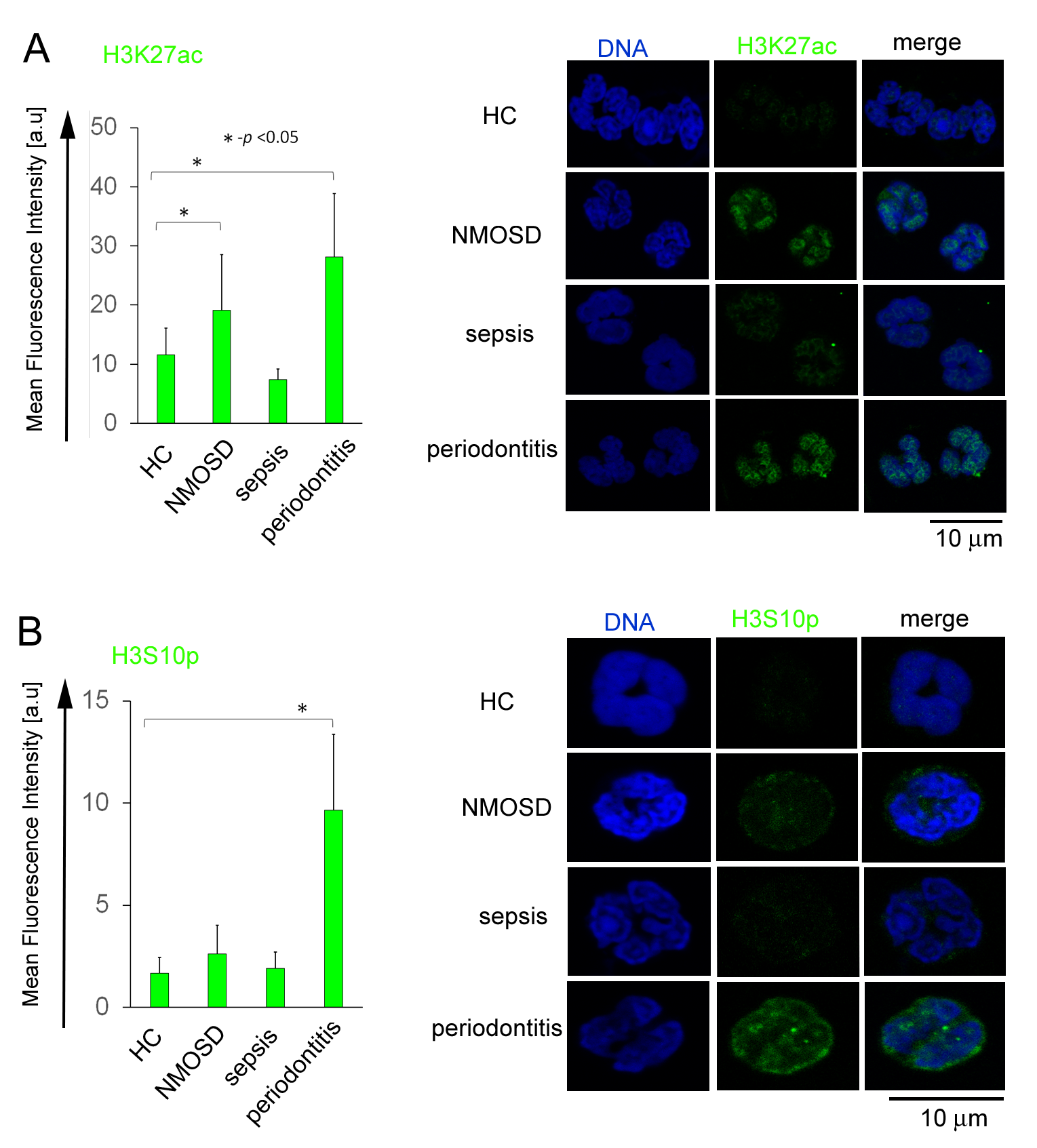

Supplement: Supplementary file 7 — Supplementary Material 7: Supplementary Figure 7. Chromatin H3K27ac marked characterised pre-activated neutrophils observed in the course of autoimmune disease such as NMOSD, while H3S10p posttranslational histone modification is one of four characteristics for polarisation of neutrophils into resolving inflammatory cells induced by IL-10, physiologically observed in periodontological patients. (A and B left panels) The Mean Fluorescence Intensity statistical comparison was calculated based on 3 cases of patients with NMOSD, 6 with sepsis and 12 with periodontitis. [file 12950_2024_389_MOESM7_ESM.tif]
