# Supplementary material for: Histone H3 posttranslational modified enzymes defined neutrophil plasticity and their vulnerability to IL-10 in the course of the inflammation
Source: J Inflamm (Lond). 2024 May 14;21:16. doi: 10.1186/s12950-024-00389-8 (PMC11095086; doi:10.1186/s12950-024-00389-8)
Supplement: Supplementary file 8 — Supplementary Material 8: Supplementary Table 1 (A, B). The list of target genes in the GO terms:‘Chromatin organisation’, ‘Histone acetyltransferase complex’, and ‘Histone methyltransferase complex’ is divided into logical subsets specific for non-, LPS, TNF, or IL-10-stimulated neutrophils. [file 12950_2024_389_MOESM8_ESM.docx]

Supplementary Tabel S1 A.


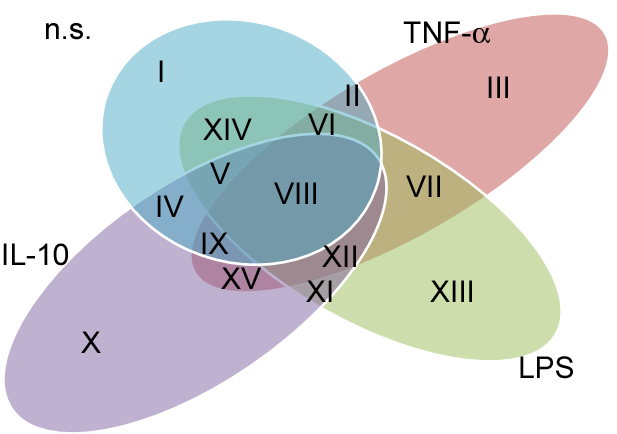


|  | GO terms |
| --- | --- |
| subsets | **Chromatin organisation (GO:0006325)** |
| I | EZH1, MYC, ATF2, H1FNT, RNF8, RNF40 |
| II | BAG6, BAZ1A |
| III | AURKA, EP300, MBTD1, MORC2, NEK11, DDB1, KMT2E, SMARCB1, MTA3, SUPT6H |
| IV | CHD3 |
| V | CXXC1, HIST2H2BE, HIRA, CHD2, SART3, DOT1L, HIST1H4F, ZBTB1, SIRT6, NTMT1, DMAP1, SETD3, NFE2, PADI4, HNRNPC, HMGA1, VPS72, ING3, ZNFX1, MBIP, BBAM2, RUVBL1 |
| VI | SS18L1 |
| VII | HIST1H3A, HIST1H2BO, CENPO, KAT6A, RBBP4, CHD1 |
| VIII | H2AFZ, INO80, KAT5, SIN3A, PHF1, PHF13, PHF19, NPM2, CREBBP, SPIN1, BRPF1, SMARCA2, EYA3, H3F3B, USP22, GRWD1, SIRT3, BRMS1L  YEATS4, BRCA2, TRRAP, SUPT4H1, BRD2, HDAC1, HDAC10, PHF2, PHF20, PHF21A, BMI1, HDAC2, BANP, HMGB1, EP400, DPY30, KAT2B,  SIRT1, SIRT5, SIRT7, PRKAA1, PRMT1, HIST1H1E, RSF1, SUPT7L, CDC73, EPC1, HDAC7, ANP32B, MAP3K12, GATAD2B, MTA2, NSD2, ARID4B, ARID1A, KDM3A, TRIM37, JADE2, KANSL3, ING5, SFMBT1, SMARCA5, SGF29, ZMIZ2, DNMT3A, MYSM1, BPTF, UBR2, SUZ12, PPM1D, RING1,  CHD7, NIPBL, TDG, KDM2A, CHD8, WAC, ACTR5, REST, RELA, SPI1, SFPQ, GPX4, SAP30, SAP30L, TAF10, KMT2D, BRD4, ENY2, KAT7, PADI2, HDAC5, TRIM28, ZMPSTE24, SUPT16H, SMARCC1, KDM4B, CENPH, PRKCEBE, H2AX, UIMC1, SKP1, NRDE2, CLOCK, PRDM2, YEATS2, CBX7, UBE2N, SRPK1, ABRAXAS1, MECP2, JMJD6, PKN1, WDR5, RBL2, EED, MIS18BP1, HIST4H4, KDM7A, SUV39H2, HUWE1, NAA50, KANSL2, NAP1L4, HAT1, MAP3K7, SMCHD1, MCRS1, BRD3, RBBP7, MSL2, EPC2, UBE2A, RSBN1, SMARCC2, CBX3, SUPT3H, CRTC2, NSD1, BAZB2, HIST1H2AC, ATXN7L3, KAT6B, ATM, HMGB2, MSL3, KNL1, SUV39H1, KDM4C, USP16, JARID2, KDM6A, UBN1, CUL4B, SUDS3, CENPX, BCOR, TAF6L, HDAC3, TET3, RBM14, CTNNB1, KANSL1, KANSL1L, ASH2L, NUCKS1, POLE3, HNRNPU, ARID4A, RYBP, PCGF5, CHD9, AEBP2, HIST2H2AA4, ASXL1, BAZ2A, PHF8, WDR82, UTP3, PBRM1, USP21, ASF1B, KDM8, CHAF1A, CHRAC1, TET2, MSL1, BRWD1, PTMA, UBE2B, BAZ1B, ACTB, RNF168, VRK1, MTF2, ACTL6A, NAA60, EMSY, DPF2, CTCF, RLF, SATB1, ARID1B, RB1, ARRB1, ZBTB7A, CBX8, MBD2, ZNF335, CTR9, HP1BP3, PAK1, ELP3, JMJD1C, SETD2, LDB1, DEK, ING2, HMG20B, RTF1, DAPK3, NAP1L1, TBL1X, TBL1XR1, CDNA1, CTBP1, SETDB1, SMARCD1, MBD1, ASH1L, ARID2, SMARCA4, NSD3, HASPIN, BAHD1, KMT5B, BRPF3, PSME4, TLK2, HIRIP3, CHD4, PRMT3, HCFC1, KDM3B, KAT8, PCGF3, TAF5, FAM172A, TDRD3, KDM1B, MBD3, USP36, JADE1, ANP32E, NCOA1, IKZF1, BRD9, CBX4, HIST1H4E, RNF20, SETD1A, RIF1, MIER3, PAGR1, PAXIP1, MORF4L1, USP3, KMT5A, PRKCD, GTF2B, DCAF1, TOP1, SPTY2D1, KDM6B, HMGN1, NASP, NR3C1, KDM5A, PPM1F, MIER1, GATAD1, RCOR1, PRMT2, PCGF1, RERE, ATAD2, SET, KMT2B, SMARCD2, ZMIZ1 |
| IX | TSPYL2, HDAC4, DTX3L, SETMAR, RBL1, H3F3A |
| X | INO80B, EHMT1, PRDM5, ZNF304, HIST1H2BJ, HIST1H2BI, MYB, ZMYND11, CABIN1, HIST1H4C, HIST1H4K, PPP5C, HIST1H2AG, HIST1H3E, IPO4, HIST2H2BF, NOC2L, HLCS, HIST1H3F, ITGB3BP, TP53, TADA1, C17orf49, HIST1HBD, HIST1H4I, HIST1H3D, APBB1, L3MBTL2, H2AFJ, H1FX, HIST1H2BH, CENPM, HMGN5, HIST1H2BN, HIST1H3H, KDM4D |
| XI | HIST1H4B, TAF5L, KDM5C, AURKB, HIST1H2BL, MCM2, PRDM7, HIST1HBE, HIST1H3I, TSPY26P, KCNQ1OT1, SMYD2, SAFB, SETD6, BABAM1, TAF9, TAF9B, TADA3, PHC1, HIST1H2BK, CDK1, SUPV3L1, PAF1, PER1, TAF1, CHEK1, KDM1A, USP7, KMT5C, USP51, HIST2H3D, SMARCAD1, SCMH1, MEN1, HIST1H2AE |
| XII | INO80C,, NPM1, CCNB1, HIST1H4H, CDK5, SMARCE1, DNMT1, TADA2A, SETD1B, BRMS1, HIST1H1D, DR1, OGT, SIRT2, PWWP3A, HIST1H1A, PRMT5, CENPS, UHRF1, ASF1A, DDB2, RCBTB1, WDR61, TSPYL4, BAP1, NCOA3, EHMT2, NCOR1, CDK2, SMYD3, ELK4, HIST1H4A, ACTR8, CHD1L, BRD8, CCNA2, KMT2A, LEO1, SATB2, H2AFV, CENPU, RCCD1, BEND3, ZNHIT1, L3MBTL1, MIS18A, RBBP5, CENPL, BCORL1, EZH2, MRGBP, PRDM4, POLE4, TAF6, ATRX, COPRS, HIST1H2AB, CARM1, ELOF1, ACTR6, CENPA, ING4, TLK1, CBX6, BRD1, KMT2C, USP15, HJURP, SIN3B, CHD6, USP49, TAF12, RIOX1, PRMT9, PWP1, RUVBL2, NAA40, KDM2B, MEAF6, PER2, OIP5, RNF2, PCGF6, DAXX, HIST1H1C, DNAJC2, SHPRH, HDAC9, H2AFY, JAK2, NUDT5, HIST1H2L, CENPC, KDM5B, HIST1H2AK, NCC2, L3MBTL3, TADA2B |
| XIII | MORF4L2, HLTF, GTF3C4, HIST1H2BC, BUD23, HIST2H2AC, SPHK2, CENPQ |
| XIV | ATXN7, CENPP |
| XV | CDKN2A, MTA1, SETD7, BRCC3, LRWD1, UB2E1, DDX11, FBL, CENPW, HIST1H4J |

Supplementary Table S1 B

|  | GO terms | |  |
| --- | --- | --- | --- |
| subsets | **Histone acetyltransferase complex (GO:0000123)** | **Histone methyltransferase complex (GO:0035097)** | |
| I | ud | ud | |
| II | ud | ud | |
| III | TAF9, | TAF9, CBX5, | |
| IV | ud | PRDM10, | |
| V | EP300, MORF4L2, DPF3, TAF9B, JADE3, TADA2B, | C17orf49, LAS1L, | |
| VI | POLE3, PHF10, | ZNF827, | |
| VII | ud | ud | |
| VIII | KAT5, TAF6L, MEAF6, CREBBP, BRD8, BRPF3, KANSL1, KANSL1L,  UBAP2L, TADA1, TAF5L, TADA2A, HCFC1, KAT8, TAF10, BRPF1,  TAF5, ZZZ3, ING4, KAT7, SUPT20H, USP22, BRD1, PHF20, TAF2, JADE1, YEATS4, MSL1, TRRAP, RUVBL1, DR1, OGT, ACTB, ACTL6A, YEATS2, EP400, KAT2B, DPF2, RSF1, WDR5, SUPT7L, EPC1, MRGBP, TADA3, MORF4L1, KANSL2, MAP3K7, DMAP1, MCRS1, MSL2, EPC2, ING3, JADE2, KANSL3, POLE4, SUPT3H, MBIP, ATXN7L3, ING5, KAT6B, TAF4, TAF7, MSL3, TAF12, RUVBL2, TAF6, SGF29, | MAX, KDM6A, KDM4A, SUZ12, CXXC1, PELP1, PHF1, PHF19, MGA, NCOA6, CHD8, KANSL1, ASH2L, RNF2, KDM5C, REST, HCFC2, KMT2A, HCFC1, KAT8, SIRT1, KMT2D, AEBP2, SETD1B, WDR82, KMT2C, PHF20, KDM4B, ZNF451, RUVBL1, MTF2, HDAC2, ZNF507, DPY30, TAF1, SETD1A, ZNF513, EZH1, WDR5, PAXIP1, PAGR1, RBBP5, EED, POGZ, HDAC9, EZH2, PRDM4, ZNF335, INO80C, E2F6, KDM6B, MCRS1, KDM5B, RBBP7, KDM5A, TEX10, MEN1, TRIM37, TAF4, KMT2B, TAF7, RUVBL2, TAF6, KDM4C, JARID2, | |
| IX | ud | ud | |
| X | ud | ud | |
| XI | ENY2, | ud | |
| XII | KAT6A | ud | |
| XIII | ud | SENP3, | |
| XIV | MSL3P1, | EPOP, | |
| XV | ud | PRMT5, RBBP4, | |

ud – undetectable
