# Supplementary material for: Histone H3 posttranslational modified enzymes defined neutrophil plasticity and their vulnerability to IL-10 in the course of the inflammation
Source: J Inflamm (Lond). 2024 May 14;21:16. doi: 10.1186/s12950-024-00389-8 (PMC11095086; doi:10.1186/s12950-024-00389-8)
Supplement: Supplementary file 9 — Supplementary Material 9: Supplementary Table 2. The list of target genes in the GO term‘Chromatin organisation’ is divided into logical subsets specific for HC, sepsis, NMOSD and periodontitis-derived neutrophils. Green font marked target genes specific for the clinic status of neutrophils (periodontitis, NMOSD and sepsis patients) and correspond with the adequate in vitro model. [file 12950_2024_389_MOESM9_ESM.docx]

Supplementary Tabel S2


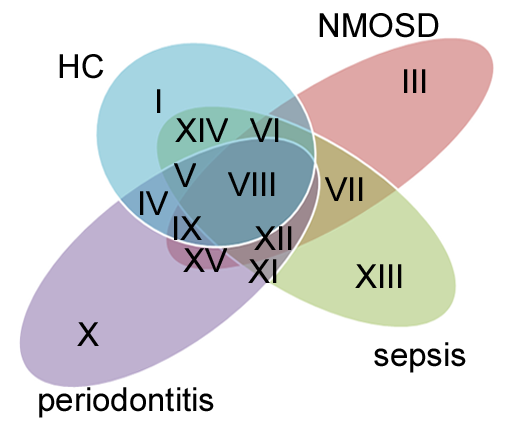

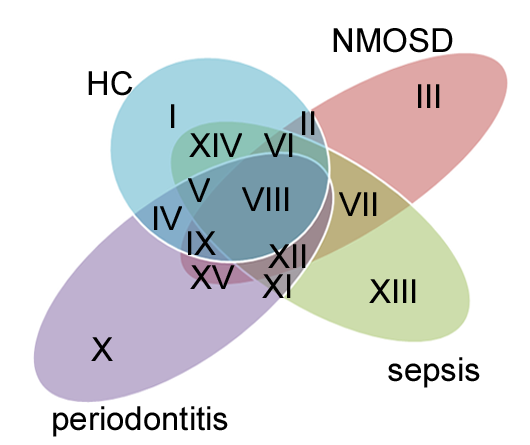

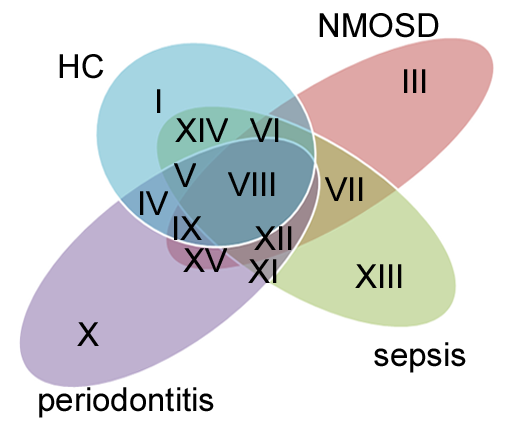


|  | GO term |
| --- | --- |
| subsets | **Chromatin organisation (GO:0006325)** |
| I | HIRA, HIST1H3E, HIST1H3H, HIST1H3B, PRDM16, CHAF1B, SMARCA1, USP51 |
| II | HIST1H4J, PABPC1L, HIST1H2BI, CBX2 |
| III | HIST1H3A, HDAC11, TAF9, AURKA |
| IV | BAP1, PRDM5, CENPQ |
| V | INO80B, MORF4L2, HIST1H3C, HMGN5, CENPL, H1F0, EP300, H3F3A, MYC, ASF1B, SPHK2, ING4 |
| VI | SMARCAD1, HIST1H2BH, HIST1H2AE, HIST1H4F, HIST1H2BF, CDKN2A, HIST1H3I, EHMT1, MBIP, TRIM16, SAFB, HIST1H3F, PPP5C, MTA1, DPF3, CENPM, EPOP, ATRX, PRDM7, ZNF304, MECON, L3MBTL2, CHEK1 |
| VII | HIST1H2AM, CDK2, PAF1, OIP5 |
| VIII | KDM4A, H2AFZ, HIST2H2BE, INO80, INO80C, NPM1, CXXC1, KAT5, PHF1, PHF13, PHF10, PHF19, MPM2, CREBBP, KDM5C, CCNB1, BRPF1, SIN3A, EZH2, SGF29, RBM14, PBRM1, SAP30, SAP30L, MAP3K7, SETD2, ATXN7L3, PAXIP1, USP3, USP36, TAF6L, SETMAR, MIS18A, EED, PRDM4, KANSL2, NAP1L4, RBBP7, MSL2, UBE2A, RSBN1, SMARCC2, POLE4, NSD1, KDM3A, ARID1A, ELOF1, PSME4, RIF1, DAXX, NASP, L3MBTL3, SETD1A, SETD3, SETD1B, SETD6, SETD7, SETDB1, SET, APBB1, NCOA1, ELP3, DNAJC2, FBL, GATAD1, NUDT5, SMARCA2, PRKCA, HIST1H2AC, PRMT9, BAZ1B, FAM172A, SMARCD1, SMARCA4, HASPIN, NSD3, NAP1L1, HIST1H4E, HIST1H3D, TBL1XR1, HIST1H2AK, HIST2H2BF, BABAM1, MRGBP, KDM2B, MIS18BP1, HIST1H2BE, HIST1H1E, HIST1H1A, SMARCE1, HIST1H2AL, HIST1H4A, ABRAXAS1, TRIM37, PRKAA1, HIST1H2BL, HIST1H4H, HIST1H4B, HIST1H1D, HIST2H2AA4, ZMPSTE24, BRMS1L, NUCKS1, HNRNPU, C17orf49, SMCHD1, SUPT16H, SMARCEC1, HMG20B, HIST1H1C, BABAM2, SPTY2D1, KDM4C, HIST1H2BK, KCNQ1OT1, HIST1H2BO, BCORL1, EPC2, MECP2, GRWD1, MCM2, SIRT3, BRMS1, YEATS4, SPIN1, MORF4L1, SUV39H1, TAF5L, HMGB2, CTNNB1, DNMT3A, CHRAC1, TRIM28, PHF21A, RPS6KA4, SUPT3H, PRKCB, PHF20, HDAC4, H2AFX, RCCD1, UIMC1, BEND3, NRDR2, PRDM2, UBE2N, CBX7, SRPK1, RBL2, KDM7A, HAT1, MCRS1, BRD3, CRTC2, SIN3B, CHD6, USP49, LDB1, RFT1, RIOX1, PWP1, RUVBL2, CDAN1, CTBP1, USP7, NAA40, ZFPM1, BAHD1, KDM1A, PER2, KMT5C, BRPF3, SIRT5, HIRP3, CHD4, TADA1, RFN2, RFN20, BRCC3, CLOCK, YEATS2, HMGA2, L3MBTL2, JMJD6, WDR5, RBBP5, HIST4H4, SUV39H2, HUWE1, GATAD2B, DNAP1, WDR61, TSPYL4, ZMYND11, MTA2, ARID4B, NCOA3, ING5, HMGB3, MYSM1, ACTR8, UBR2, CHD1L, PPM1D, TDG, PADI4, WAC, REST, RELA, SPI1, SFPQ, TAF10, KMT2D, KAT7, HDAC5, KDM4B, CENPH, SKP1, OGT, HDAC1, PHF2, ZBTB1, BANP, HMGB1, EP400,  KAT2B, PWWP3A, GTF2B, DCAF1, NR3C1, PPM1F, PCGF1, TAF12, TADA2A, DNMT1, BAZ2B, HMGA1, NTMT1, UHRF1, ASF1A, RCBTB1, DDB2, NSD2, KANSL3, JADE2, GTF3C4, SFMBT1, NCOR1, SMARC5, SMYD3, ZMIZ2, ELK4, BPTF, SUZ12, RING1, NIPBL, CHD7, NEF2, BRD8, CCNA2, KDM2A, CHD8, ACTR5, KMT2A, GPX4, BRD4, LEO1, PADI2, HNRNPC, SATB2, SMYD2, H2AFV, CENPU, KAT6B, ATM, IPO4, JARID2, USP16, KDM6A, UBN1, CENPX, BCOR, COPRS, SMARCB1, KANSL1, KANSLK1L, ZBTB7A, RB1, ARRB1, CBX8, MBD2, VPS72, CTR9, PAK1, PRMT3, KAT8, SIRT1, HCFC1, KDM3D, PCGF3, TDRD3, KDM1D, RUVBL1, RBL1, DDX11, PAGR1, EYA3, H3F3B, AURKB, USP22, DOT1L, TRRAP, BRD2, BMI1, HDAC2, DPY30, SUBT7L, EPC1, HDAC7, ASH2L, CARM1, ARID4A, ACTR6, CENPA, ASXL1, SIRT6, WDR82, GRD1, KMT2, KDM8, CHAF1A, BRWD1, HLCS, PTMA, LRWD1, ACTL6A, MTF2, EMSY, TAF1, DPF2, SATD1, ZNF335, HP1BP3, JMJD1C, ING2, DEK, ING3, UB2E1, TBL1X, MBD1, ASH1L, MEAF6, KMT5B, TLK2, TAF5, PCGF6, MBD3, JADE1, ANP32E, IKZF1, BRD9, CBX4, SHPRH, H2AFJ, MIER3, HDAC9, KMT5A, PRKCD, H2AFY, JAK2, TOP1, KDM6B, CENPW, HMGN1, CENPC, KDM5B, KDM5A, MIER1, SCMH1, RCOR1, PRMT2, MEN1, RERE, ATAD2, NACC2, TADA2B, KMT2B, SMARCD2, ZMIZ1, MSL3, KNL1, PHC1, CUL4B, SUDS3, HDAC6, TET3, NOC2L, POLE3, RYBP, CHD9, PCGF5, AEBP2, BAZ2A, PHF8, TLK1, CBX6, UTP3, USP21, SUBV3L1, TET2, MSL1, CHD3, PER1, UB2B, ACTB, RNF168, VRK1, NAA60, USP15, HJURP, CTCF, RLF, ARID1B |
| IX | JADE3, TAF6, TADA3, ITGB3BP, PKN1, ATF2, HDAC10, CABIN1, PYL2, TAF9B, DAPK3, CENPV |
| X | ZNFX1, HIST2H2AC, BUD23, KDM4D |
| XI | ATXN7, RPS6KA5, KMT2E |
| XII | CDK5, HIST2H3D, H1FX, CHD2, SART3, DTX3L, NAA50, SIRT2 |
| XIII | HLTF, SETD5, HIST1H2AG, HIST3H2A,HIST1H2AI, WDR5B, HIST1H2AB, HIST2H2AB, HIST1H2AH, TSPY26P, HIST1H2BG, SOX9, SMARCD3, ZNHIT1, H1FNT, CENPI, KDM5D, FOXA1, PHB, TP53, SS18L1, RBBP4, HIST1H4C, MTHFR, HIST1H2BJ |
| XIV | MTA3, HIST1H2BD, HIST1H4I, SIRT4, SOX2, PRMT6, RNF8, BAG6, HIST1H4K, L3MBTL4, ENY2, KAT6A, IRF4, CHD1, CDK1 |
| XV | ARID2 |
